# WERF Endometriosis Phenome and Biobanking Harmonisation Project for Experimental Models in Endometriosis Research (EPHect-EM-Heterologous): heterologous rodent models

**DOI:** 10.1093/molehr/gaaf022

**Published:** 2025-07-09

**Authors:** M Louise Hull, Raul Gomez, Warren B Nothnick, Ruth Gruemmer, Katherine A Burns, Mohammed Zahied Johan, Isabella R Land, Stacey A Missmer, Lone Hummelshoj, Erin Greaves, Kaylon L Bruner-Tran, for the EPHect Experimental Models Working Group, Nick A Andrews, Michael S Anglesio, Caroline B Appleyard, Joe Arosh, Christian M Becker, Kaylon L Bruner-Tran, Katherine A Burns, Ronald L Chandler, Julie A Christianson, Fiona L Cousins, Kelsi N Dodds, Victor Fattori, Asgi Fazleabas, Caroline Gargett, Juan S Gnecco, Raul Gomez, Martin Götte, Erin Greaves, Linda G Griffith, Patrick G Groothuis, Ruth Grümmer, Sun-Wei Guo, Shannon M Hawkins, M Louise Hull, Lone Hummelshoj, Mark Hutchinson, Mohamed Gamal Ibrahim, Elizabeth E Marr, Stacy L McAllister, Stacey A Missmer, Jeffrey Mogill, Jens Nagel, Warren B Nothnick, Paulina Nunez-Badinez, Kevin G Osteen, Daniëlle Peterse, Michael S Rogers, Andrea Romano, Philippa T K Saunders, Miguel Ángel Tejada, Kathy L Sharpe-Timms, Waldiceu A Verri, Paola Viganó, Katy Vincent

**Affiliations:** The Robinson Research Institute, University of Adelaide, Adelaide, Australia; Research Unit on Women's Health-INCLIVA, Institute of Health Research, Valencia, Spain; Department of Pathology, University of Valencia, Valencia, Spain; Department of Cell Biology and Physiology, Center for Reproductive Sciences, University of Kansas Medical Center, Kansas City, KS, USA; Department of Anatomy, University Hospital Essen, University of Duisburg-Essen, Essen, Germany; Department of Environmental and Public Health Servies, University of Cincinnati College of Medicine, Cincinnati, OH, USA; Centre for Cancer Biology, University of Adelaide, Adelaide, Australia; Department of Obstetrics and Gynecology, Vanderbilt University Medical Center, Nashville, TN, USA; Department of Obstetrics and Gynaecology, University of Michigan, Ann Arbour, MI, USA; Department of Epidemiology, Harvard TH Chan School of Public Health, Boston, MA, USA; World Endometriosis Research Foundation, London, UK; World Endometriosis Research Foundation, London, UK; World Endometriosis Research Foundation, London, UK; Division of Biomedical Sciences, Warwick Medical School, University of Warwick, Coventry, UK; Department of Obstetrics and Gynecology, Vanderbilt University Medical Center, Nashville, TN, USA; World Endometriosis Research Foundation, London, UK

**Keywords:** endometriosis, experimental models, rodents, heterologous, research, collaboration

## Abstract

Endometriosis, defined as the growth of endometrial-like tissues outside the uterus, is a common disease among women. Numerous *in vivo* rodent models of endometriosis have been developed to explore multiple aspects of this poorly understood disease. Heterologous models utilize human endometrial tissues engrafted into immunocompromized mice, while homologous models engraft rodent endometrium into immunocompetent mice or rats. Heterologous models of endometriosis more closely replicate the human disease; however, the murine humoral immune response must be suppressed to prevent rejection of the xenograft tissue. Although the innate immune system remains intact, suppression of the humoral response leads to a markedly different local and systemic immune environments compared to humans. Despite this limitation, experiments using heterologous models have contributed significantly to our understanding of endometriosis establishment and progression, the pre-clinical effectiveness of various therapeutic strategies, and genetically modifiable host factors that contribute to disease. Unfortunately, a lack of harmonization of the models used by different laboratories has impeded the reproducibility and comparability of results between groups. Therefore, the World Endometriosis Research Foundation (WERF) formed an international working group of experts in heterologous models of endometriosis to develop guidelines and protocols that could contribute to unifying experimental approaches across laboratories. Nine critical variables were identified: (i) mouse strain; (ii) human tissue type; (iii) hormonal status of the human tissue donor; (iv) human tissue preparation; (v) method and location of tissue placement; (vi) hormonal status of the recipient animal; (vii) whether or not mice were engrafted with human immune cells; (viii) endpoint assessments; and (ix) number and type of replicates. Herein, we outline important considerations for each major variable and make recommendations for unification of approaches. Widespread adoption of harmonized protocols and implementation of standardized documentation and reporting should further improve the reproducibility and translation of experimental findings both within and between laboratories.

## Introduction

Endometriosis affects an estimated 190 million women worldwide, leading to a significant personal and societal burden due to its painful and fertility-related symptoms (Nnoaham *et al.*, 2011; Zondervan *et al.*, 2020). There is no known cure, and treatments are associated with low long-term success rates and significant side effects (Johnson *et al.*, 2013).

Human endometriosis is defined as endometrial-like tissue outside the uterus; however, this definition does not encompass the complex symptomatic, pathobiologic, and multisystemic nature of the disease (Zondervan *et al.*, 2020). The overwhelming prevalence of endometriosis and lack of known aetiology highlight the need for a better understanding of the basic biology of endometriosis formation and persistence to enable subsequent identification of targets for effective therapies.

The cellular and structural composition of endometriotic lesions can vary widely. Lesions may have abundant or scant numbers of endometrial-like stromal cells, the presence or absence of epithelial glands, and, often, haemorrhagic deposits (Clement, 2007; Nnoaham *et al.*, 2011; Vigano *et al.*, 2018). The histological appearance of lesions also varies depending on different contributions of extracellular matrix, whether or not fibrosis is present (Li *et al.*, 2022), synchronicity of the lesions with the menstrual cycle (Colgrave *et al.*, 2020), and the extent of immune and nerve cell infiltration (Tran *et al.*, 2009). Survival of endometriosis lesions requires vascular growth into the tissue. Vascularization, in addition to providing the needed blood supply, also enables an influx of immune cells, which aids in lesion innervation and the development of an inflammatory microenvironment (Greaves *et al.*, 2017; Forster *et al.*, 2019; Hogg *et al.*, 2020, 2021; Panir *et al.*, 2022).

Endogenous production of steroids (oestrogen and progesterone) also significantly impacts the survival and function of ectopic endometrial-like cells (Saunders and Horne, 2021). Endometriosis has long been called an oestrogen-dependent disease due to the contribution of this steroid to neuroangiogenesis and the development of neuroinflammation (Greaves *et al.*, 2014, 2015). Thus, the local hormone microenvironment, which can vary widely between lesions, likely contributes to not only the observed differences in disease presentation and colour but also the tissue responses to treatment (Zondervan *et al.*, 2020).

Endometriosis occurs naturally only in humans, non-human primates, and other, rare, menstruating mammals. For this reason, scientists have developed the use of both heterologous and homologous rodent models in order to study this disease. Rodent models of endometriosis have been found to be useful for studying how various environmental, hormonal, genetic, or therapeutic factors promote or inhibit disease development (Bruner-Tran *et al.*, 2018; Burns *et al.*, 2021). Heterologous mouse models use human tissues to establish experimental disease in immunocompromized mice, while homologous models use uterine tissue from the same species to establish ectopic disease. Injected human or murine tissues are amenable to the use of fluorescent proteins, which can aid in the identification of small lesions as well as to track lesion size (Fortin *et al.*, 2004; Hirata *et al.*, 2005).

Heterologous mouse models are unique in their ability to assess the potential for different types of human endometrium and endometriotic tissue to engraft at an ectopic site. To our knowledge, no studies have reported the engraftment of human endometrial tissues into rats for the study of endometriosis. The earliest studies using heterologous models of experimental endometriosis were conducted using Hsd:Athymic Nude*-Foxn1^nu^* (athymic nude or, simply, nude) mice. More recent studies using mice with Severe Combined Immunodeficiency (CB17/IcrHanHsd-Prkdc*^scid^/Ozarc* (congenic SCID)) have also been published (Pearson *et al.*, 2008). These mice are often crossed to a non-obese diabetic background to create NOD.CB17-Prkdc^*scid*^/Ozarc (NOD-SCID) mice. Also commonly used are the Recombinant Activating Gene 2/common cytokine receptor γ chain (c) double null mice (C57BL/6NTac.Cg-*Rag2^tm1Fwa^ Il2rg^tm1Wjl^* (Rag2γ(c)) (Mian *et al.*, 2020).

While all four strains accept human tissues, as discussed below, SCID, NOD-SCID and Rag2γ(c) mice additionally allow adoptive transfer of human immune cells. Numerous other immunocompromized mouse strains have been described for human cancer studies and may also have utility for endometriosis-related investigation (Mian *et al.*, 2020; Yang, 2021).

The first experimental endometriosis study using human tissues in immunocompromized mice was published in 1984 (Zamah *et al.*, 1984). In this study, eutopic endometrium from women with or without endometriosis or endometriotic tissues obtained from women with the disease were introduced into the peritoneal space of three groups of nude mice, half of which were supplemented with oestrogen. While experimental lesions developed in most mice, those receiving oestrogen and injected with ectopic lesions exhibited more extensive disease. A year later, Bergqvist *et al.* (1985) published a similar study in which they examined the impact of exogenous hormones on the ability of human endometrial tissues to establish lesions in nude mice. Eutopic endometrium from women with or without endometriosis or endometriotic lesions from patients was introduced into the peritoneal cavity of nude mice. All mice were initially given oestrogen to promote lesion establishment. Mice were then divided into groups that were given oestrogen only or oestrogen with either medroxyprogesterone acetate or danazol. At necropsy, gross and histologic assessment indicated that hormonal variations in the host peritoneal environment primarily influenced lesion development rather than differences between endometrium and endometriotic tissues. These early studies using ‘humanised’ models of experimental endometriosis demonstrated key concepts and understanding around the basic science of endometriosis and demonstrated the distinctive ability of heterologous mouse models to independently investigate the contributions of donor endometrial tissue and the host site response. Specifically, the ability to unravel disease-specific adaptations in the endometrium and immune responses is key and cannot be performed in homologous models. For example, Bruner-Tran *et al.* (2008) demonstrated that subcutaneously placed eutopic endometrium from human donors with endometriosis led to more numerous ectopic lesions that were larger and more highly vascularized compared to mice provided with tissues from a healthy donor. Experimental lesions established by endometrium from donors with and without endometriosis also exhibited differential responses to progesterone (Bruner-Tran *et al.*, 2002; Palmer *et al.*, 2016).

More recent studies have implicated a role for innate host immune responses in influencing the establishment or progression of disease. For example, a longitudinal assessment of heterologous lesion development (Johan *et al.*, 2019) revealed that pro-inflammatory macrophages were present 7 days after tissue implantation within the periphery of lesions displaying central necrosis. By Day 14, pro-repair macrophages were identified more centrally in ectopic endometriosis-like lesions that displayed remodelling of the glands and stroma. Species-specific immunohistochemistry and histochemical stains previously determined the predominantly murine origin of these macrophages, which was confirmed in a species-specific transcriptomic analysis (Hull *et al.*, 2008). Such studies, which have significantly expanded our understanding of lesion formation, can only be performed in xenograft models.

As stated above, SCID and Rag2γ(c) mice can be engrafted with both human tissues and human immune cells and are useful for exploring the role of the immune response in lesion development. Bruner-Tran *et al.* (2010) reported 100% of Rag2γ(c) mice injected only with endometrial tissues from disease-free donors exhibited experimental disease. However, engraftment with both endometrial tissues and immune cells from disease-free donors led to a significant reduction in both the percentage of mice with lesions and the overall disease burden. In contrast, engrafting immune cells obtained from women with endometriosis into mice bearing ectopic lesions derived from endometrium from healthy women was associated with larger and more numerous lesions (Bruner-Tran *et al.*, 2018). These data suggest that human immune cells from women without endometriosis protect against the development of experimental disease, whereas immune cells from women with endometriosis promote disease.

Heterologous models can also compartmentalize host and endometrial tissue exposures to environmental factors that influence endometriotic disease establishment. Investigators have used this method to determine which compartment to target to disrupt host–endometrial crosstalk in order to suppress lesion development (Hull *et al.*, 2012). Exposure of human endometrial tissues to environmental toxicants such as dioxin (Bruner-Tran *et al.*, 2008), inflammatory cytokines (Stocks *et al.*, 2017), or growth factors in seminal plasma (McGuane *et al.*, 2015) prior to injection into mice can aid the delineation of tissue factors that contribute to lesion development. Similarly, the recipient animal can also be exposed to dietary factors, environmental toxicants, inflammatory cytokines, or a myriad of other agents prior to the introduction of tissue (Greenberg and Slayden, 2004; Krikun *et al.*, 2010; Herington *et al.*, 2013; Rudzitis-Auth *et al.*, 2013; Stocks *et al.*, 2017). These types of studies are critically important in determining the role of each tissue compartment in disease development.

As heterologous models use patient-derived human tissues, they are also valuable for the preclinical assessment of potential therapeutics. Phase 1 pharmaceutical trials using heterologous mouse models of endometriosis are a cost-effective way of screening potential therapies for safety and efficacy against human tissues prior to conducting similar studies in non-human primates or clinical trials in women (Bruner-Tran *et al.*, 2009; Palmer *et al.*, 2016). Although beyond the scope of this article, heterologous models are unique in that they enable experimental genetic expression or deficiency solely within the host compartment (Capobianco *et al.*, 2011). Heterologous studies as described herein are especially well-positioned to create a better understanding of exposures that promote or exacerbate endometriosis and/or identify strategies to prevent this disease.

Previously, the World Endometriosis Research Foundation (WERF) Endometriosis Phenome and Biobanking Harmonisation Project (EPHect) established standard recommendations and minimum requirements for the collection of clinical and surgical data from those with endometriosis (Becker *et al.*, 2014; Vitonis *et al.*, 2014), as well as a standardized physical examination assessment in endometriosis (Lin *et al.*, 2024). WERF also developed internationally agreed upon standard operating procedures (SOPs) for the collection, processing, and storage of tissue and fluid biospecimens (Becker *et al.*, 2014; Fassbender *et al.*, 2014; Rahmioglu *et al.*, 2014; Vitonis *et al.*, 2014). Through this process, WERF identified an unmet need to develop standards of practice in experimental models of endometriosis.

Given the broad utility of human–mouse xenograft models, the heterologous working group was tasked with developing recommendations to harmonize procedures and to create reporting standards for published studies. A review of the literature identified nine major procedural variations across studies employing heterologous mouse models for endometriosis research. These were: (i) the strain of mouse selected; (ii) the type of human endometrial tissue utilized; (iii) hormonal status of human tissue donor; (iv) preparation of tissues prior to introduction into mice; (v) method of placement and ectopic location of endometrial tissue in mice; (vi) hormonal status of the recipient animal; (vii) whether or not mice were additionally engrafted with human immune cells; (viii) the endpoint assessments; and (ix) the number and type of replicates. Additionally, we found that the rationale for a given experimental design choice was often poorly described, and many papers lacked essential information that impeded interpretation of the data and/or the reproducibility of the study.

Although no *in vitro* or *in vivo* model can successfully recreate all aspects of human endometriosis, identifying the primary question of interest enables the selection of the most relevant model. Such a ‘best-fit’ approach was recently described for the use of homologous endometriosis models (Burns *et al.*, 2021) and is also applicable to xenograft models. Herein, we present recommendations and considerations for experimental design when using heterologous mouse models. A decision tree was created to aid investigators during the initial stages of planning their experiments. These recommendations are not intended to restrict scientific discovery. Instead, these recommendations should enhance future developments in novel heterologous mouse models that will further improve our understanding of endometriosis.

Recommendations and SOPs were also developed for homologous rodent models, endometriosis organoids, and pain behaviour, and the results are presented in the associated WERF companion papers (Burns *et al.*, 2025; Dodds *et al.*, 2025; Marr *et al.*, 2025).

## Methods

The EPHect Experimental Models Working Group was established following the WERF Experimental Models workshop, which was held virtually in 2021. Internationally recognized researchers with expertise in experimental endometriosis were invited to attend the workshop, provided they had contributed to the field by having conducted research using specific rodent model(s). Additional criteria included (i) having published manuscripts with evidence of comprehensive histological analysis, (ii) having exemplary experimental designs, and (iii) having published at least three peer-reviewed research studies using the same model. Publications were identified using the search words: ‘endometriosis’ + ‘mouse’ + ‘murine’ + ‘rat’. The manuscript requirement was less stringent for those working with emerging models of endometriosis. Of the 44 experts who were invited to participate, 39 accepted. Also included as observers were six individuals who were students or in the early stages of their careers. Eight working group members were added after the workshop, and three left the group shortly thereafter, leaving a total of 44 individuals who ultimately contributed to the manuscripts.

The aims of the workshops were to reappraise the current and emerging models of endometriosis and to develop protocols and criteria (SOPs) that meet a minimum set of standards and to produce a decision tree based on the objective(s) of a given study. Since no single model can replicate all parameters of endometriosis, it was clear that one set of standards or SOPs would be inadequate. Thus, at the conclusion of the workshops, four working groups were established, and consortium members were invited to join one or more groups based on their interest and experience (Supplementary Fig. S1). The four groups were: (i) heterologous models, (ii) homologous models, (iii) pain behaviour, and (iv) organoids as an emerging (*in vitro*) model for endometriosis. For each group, a discussion forum was utilized to submit SOPs for circulation and standardization, and to enable a platform for discussions. Further discussions were held between authors to continue dialogues and develop consensus on areas for unification.

The heterologous working group conducted online searches within PubMed using the terms ‘xenograft mouse endometriosis’ (55 results), ‘endometrial human mouse lesion tissue endometriosis’ (106 results), and ‘mouse model of endometriosis heterologous’ (19 results). Studies specific to ovarian cancer were removed, leaving a total of 165 papers for further consideration. Additional searches using similar search terms with ‘rat’ instead of ‘mouse’ did not return any appropriate studies. All papers were reviewed for content, experimental design, and experimental details. In addition to the nine variables mentioned above, we identified four major experimental decisions that must be made prior to study initiation.

Thus, 17 detailed protocols were received from members of the heterologous working group. Collectively, these protocols contained all necessary steps to establish human endometrial tissues as ectopic lesions in immunocompromized mice with or without adoptive transfer of human immune cells. All protocols were circulated, and each member of the working group added suggestions and recommendations for clarity or additional details that may benefit new investigators. Each protocol was reviewed, revised for clarity, and, when necessary, additional details were elicited and added. Similar protocols were unified and combined. A detailed SOP for documentation was also developed using a generic online template provided by Parker *et al.* (2015) at McMasters University. As no single model is appropriate for every research scenario, these protocols formed the basis of a decision tree to aid study design for future studies.

## Results

The working group reviewed more than 165 published papers that used heterologous models of experimental endometriosis. The majority of studies used athymic nude mice, while the remaining studies chose SCID, NOD-SCID, or Rag2γ(c) mice. Other common model variations included the type, source, and cycle phase of human tissues, the hormonal status of the recipient animal, the method of experimental disease induction, and the location of tissue placement. Only a few studies that additionally engrafted human immune cells were found. Finally, we found a lack of unity with regard to endpoint assessments and the number and type of experimental replicates.

**Figure 1.**
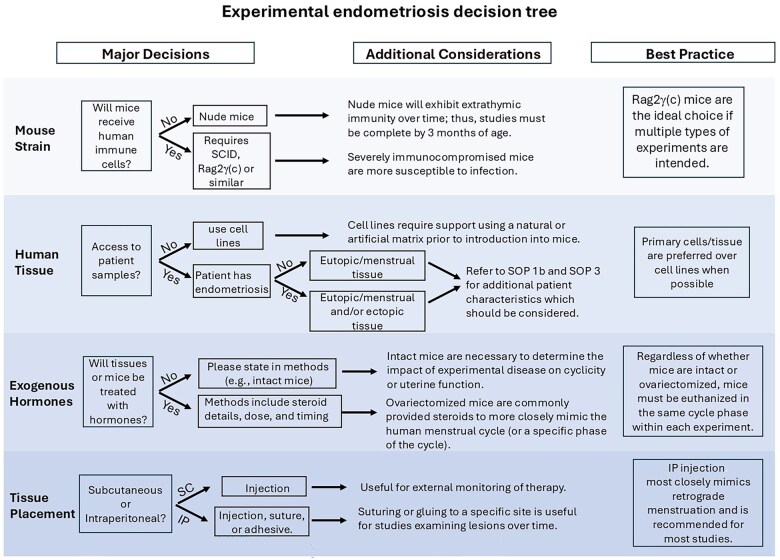
**Decision tree**. There are four major design variables that must be addressed prior to the initiation of a heterologous experimental endometriosis study. These variables are presented along with experimental considerations, which should aid in decision-making.

We developed a flowchart-style decision tree to aid investigators in developing the best strategy for their study question (Fig. 1). We also produced 15 SOPs (Supplementary File S1), which detail all the necessary steps for establishing and documenting experimental human endometriosis in immunocompromized mice. These are referenced below, as appropriate.

### Major decisions to be made prior to study initiation

#### Choice of recipient strain

**Figure 2.**
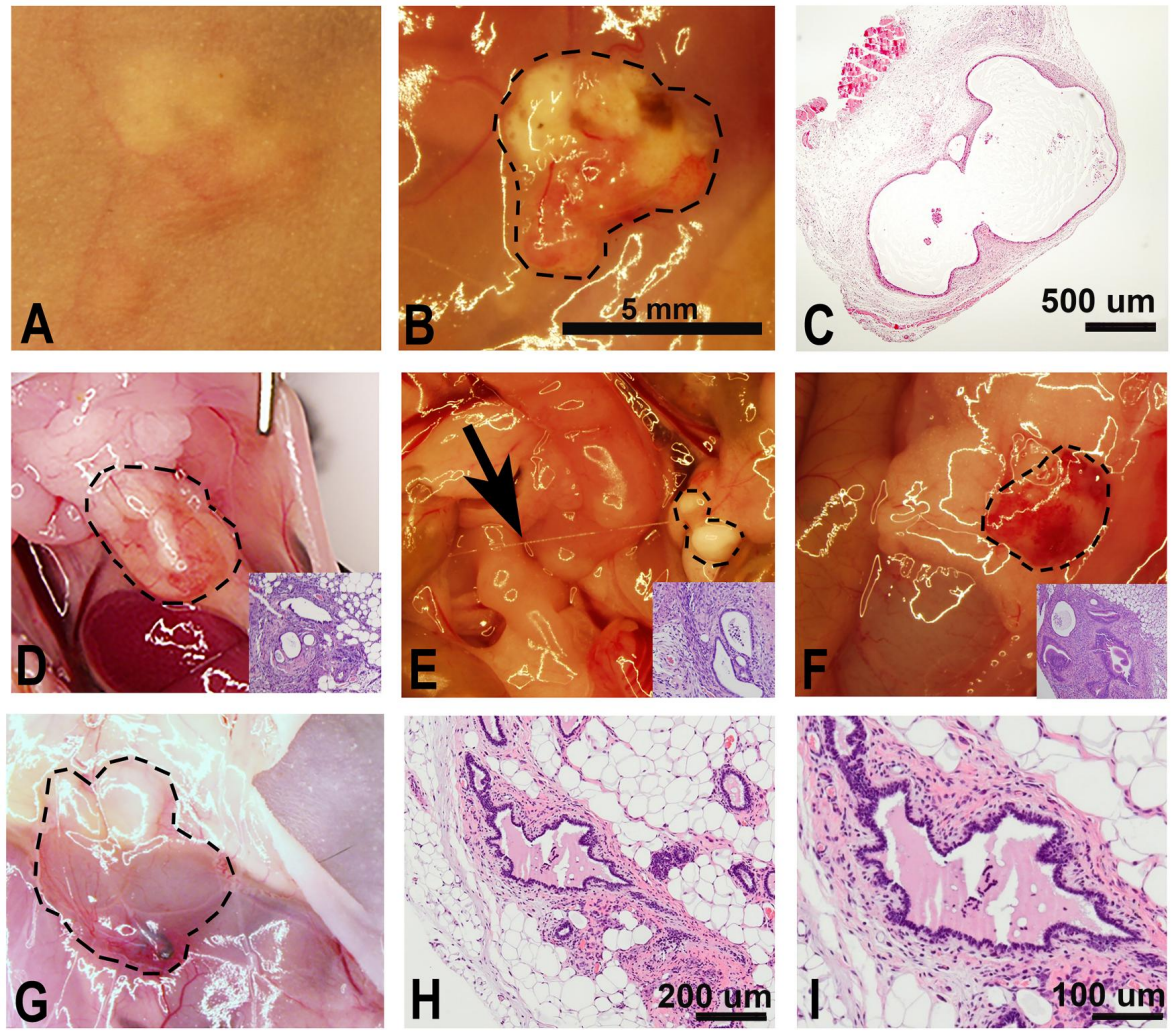
**Experimental human endometriosis established in immunocompromized mice**. (**A**) Subcutaneous lesion observed through the skin of a nude mouse. (**B**) Lesion in (A) with skin removed (15×). (**C**): Haematoxylin and eosin (H&E) staining of lesion in A and B (40×). (**D**–**F**) Gross morphology of common types of intraperitoneal lesions. A thin adhesion is also visible in (E) (arrow). Insets: H&E of the same lesion, 100×. (**G**) Gross morphology of a lesion created using isolated cells embedded in a hydrogel matrix. (**H** and **I**) H&E staining of lesion in G. 100× and 200×, respectively.

**Figure 3.**
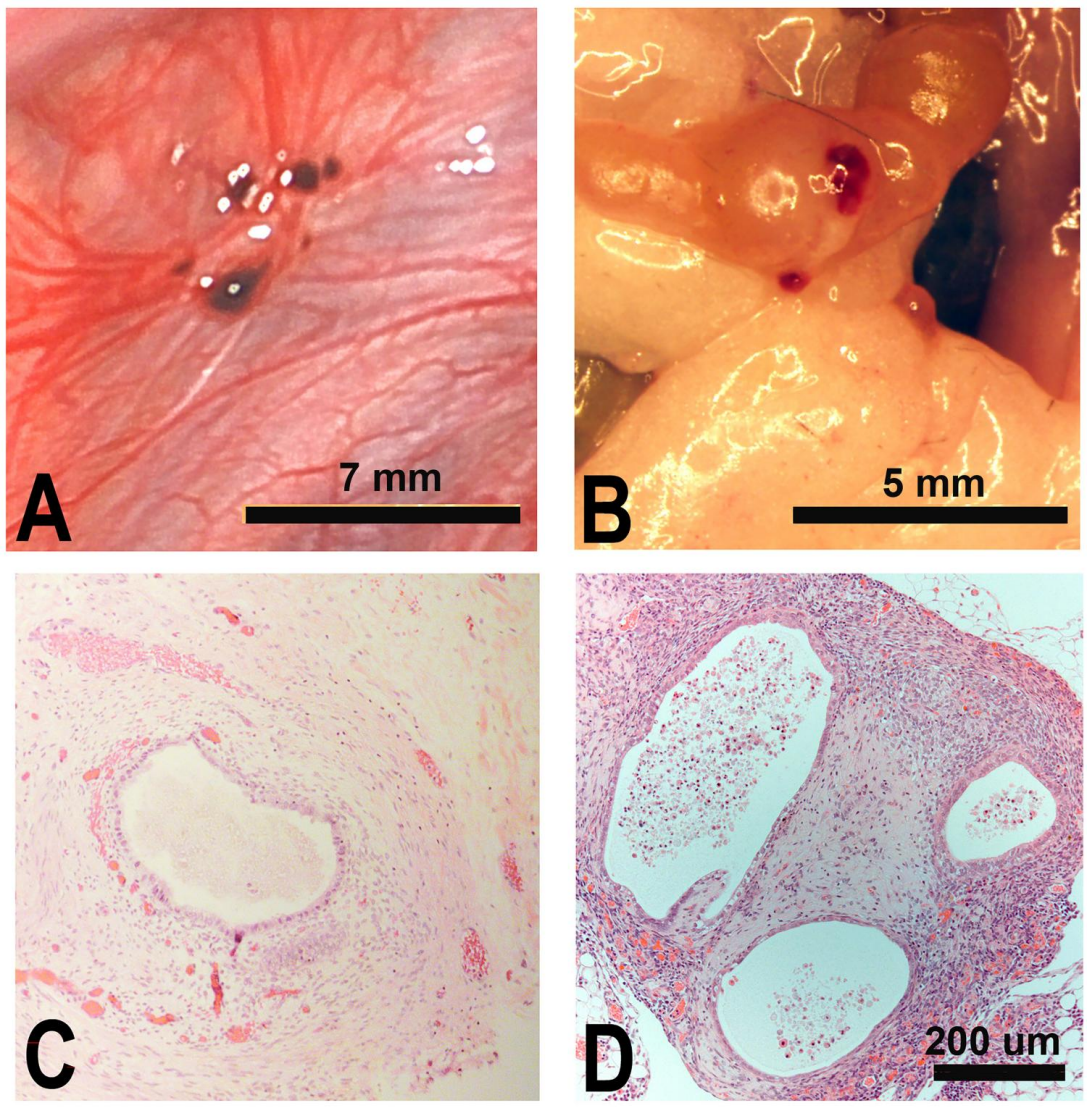
**Endometriotic lesions: native human disease versus experimental model**. Lesions of human endometriosis, photographed during a laparoscopic surgery (Courtesy of Dr Gil Wilshire) often appear as highly vascularized red nodules (**A**). Human tissues growing as ectopic lesions in nude mice frequently exhibit a similar phenotype (**B**). The histology of formalin fixed, paraffin-embedded human endometriotic lesions (**C**) and human tissues removed from mice as lesions (**D**) and stained with haematoxylin and eosin, also frequently exhibit marked similarities. Original magnification, 100× for both photomicrographs.

As stated above, the establishment of human tissues in mice requires the use of animals with limited adaptive immunity in order to avoid rejection of tissue. Of the papers reviewed by the consortium, the most commonly selected mouse strains were athymic nude, SCID, NOD-SCID, and Rag2γ(c). Despite their immunocompromized state, the innate immune system is intact and can interact with introduced human endometrial tissue in a manner that permits ectopic endometriosis-like lesion development. The ability of SCID, NOD-SCID, and Rag2γ(c) mice to accept both human tissues and immune cells makes these animals extremely useful for endometriosis studies investigating the role of both tissue compartments in disease pathogenesis. Regardless of the strain, experimental endometriotic lesions exhibit a wide range of phenotypes, which may or may not be well-vascularized (see Fig. 2 for examples). Adhesions are also frequently observed (Fig. 2E, arrow). The histology of the lesions is markedly similar to the human disease, both grossly and histologically (Fig. 3).

Use of any immunocompromized mouse strain requires housing in a pathogen-free environment with sterile food and water. All handling should be conducted within a laminar flow hood, and, if surgery is required, aseptic conditions are necessary. However, compared to nude mice, more severely compromized mouse strains are more susceptible to infection. Thus, in the absence of introduced human immune cells, an investigator must weigh the pros and cons of using more severely immunocompromized mouse strains (Table 1).

**Table 1.** Overview of the characteristics of selected immunocompromized mouse strains.

	Athymic nude	Congenic SCID	Rag2γ(c)
**Formal gene nomenclature** (may vary slightly with vendor)	Hsd: Athymic nude-Foxn1^nu^	C.B-17/IcrHanHsd-Prkdc^scid^	C57BL/6NTac.Cg-Rag2^tm1Fwa^ Il2rg^tm1Wjl^
**Genetic origin**	Spontaneous mutation of *Foxn1* on Chromosome 11.	Spontaneous autosomal recessive mutation of *Prkdc* (DNA repair enzyme) on Chromosome 16.	Targeted double mutation obtained by crossing gamma c knockout and Rag2 deficient mice (C57Bl/6 background).
**Development or discovery**	Discovered in 1962 in Virus Laboratory, Ruchill Hospital, Glasgow, Scotland.	Developed in 1980 by Bosma and associates in BALB/*c-Igh*^*b*^ mice at Fox Chase Cancer Center.	Developed by Mazurier and colleagues in 1999 at Université Victor Segalen, Bordeaux, France.
**Lacking T lymphocytes**	Yes	Yes	Yes
**Lacking B lymphocytes**	No (impaired function)	Yes	Yes
**Lacking natural killer cells**	No	No	Yes
**Lacking macrophages**	No	No	No
**Lacking PMN**	No	No	No
**Develop extra-thymic immunity**	Yes	Yes	No
**Lifespan**	8.5 months–1 year	Compromised due to development of lethal thymomas.	1 year+
**Effects on reproduction**	Homozygous females exhibit underdeveloped mammary glands (do not lactate).	Can be limited by shorter life span	None Known
**Ability to perform long-term (>3 months) xenograft studies**	No	No	Yes
**Human endometrial xenograft survival**	Up to 21 days, ‘take-rate’ of 60%	Up to 28 days; ‘take-rate’ of up to 100%	Up to 112 days; ‘take-rate’ of up to 100%
**Accept human immune cell engraftment**	No	Yes	Yes
**Pros**	–Widely available –Hairless –Docile and easy to work with	–Readily propagated by breeding with no known fertility defects –Accept human immune cells	–Readily propagated by breeding with no known fertility defects –Accept human immune cells
**Cons**	–Development of extrathymic immunity –Homogenous dams do not lactate.	–Limited availability of mice –Development of extrathymic immunity –Susceptible to infection	−Breeding restricted by the vendor (Taconic Biosciences)

PMN, polymorphonuclear leukocyte

##### Nude mice

Athymic nude mice were first identified in the Virus Laboratory at Ruchill Hospital in Scotland in 1962. These outbred albino mice developed a spontaneous mutation in the forkhead box N1 (*Foxn1*) gene, which is associated with thymic dysgenesis (Flanagan, 1966; Pantelouris, 1968). Consequentially, these mice exhibit an abnormally low number of T cells, impairing humoral immunity. Although homozygous nude mice have a normal complement of B cells, their lack of T cells prevents complete B-cell maturation (Table 1). Thus, antibody formation, cell-mediated responses, delayed hypersensitivity, and graft rejection are all suppressed in nude mice. At around 3–4 months of age, nude mice develop extra-thymic humoral immune responses (MacDonald *et al.*, 1986), which leads to human tissue rejection. For this reason, experimental studies must be completed by 4 months of age. However, nude mice are an ideal choice for short-term studies that do not require the adoptive transfer of human immune cells. Because of their docile nature and lack of hair, which simplifies surgical procedures such as ovariectomy and endometrial tissue injection, these mice are easy to work with and a good choice for less experienced researchers and students. Subcutaneously placed lesions can also be directly visualized in live mice making these animals especially useful for monitoring response to therapy (Fig. 2A–C). However, maintaining a colony of nude mice is difficult as homozygous females do not lactate and require pups to be cross-fostered for their survival.

##### SCID mice

SCID is a genetic condition characterized by absent or dysfunctional T and B lymphocytes in humans and mice. The humoral immune system is severely compromized and unable to mount an adaptive immune response to pathogens (de Villartay, 2009). In mice, the condition commonly results from a recessive mutation, which limits the activity of the DNA repair enzyme protein kinase, DNA activated, catalytic polypeptide (*Prkdc^−/−^*). This mutation was first described in BALB/c-*Igh^b^* mice, which had inadequate cellular and humoral adaptive immune responses (Bosma *et al.*, 1980). Several other gene mutations have been identified to result in a SCID phenotype, and in some cases, the mice also show deficits in natural killer (NK) cells and other lymphocytes (reviewed in Chen *et al.*, 2022). Like nudes, some SCID strains exhibit an age-related compensatory immunity, which limits the duration of studies that can be conducted.

Aoki *et al.* (1994) longitudinally compared lesion development in SCID and nude heterologous mouse models of endometriosis. Human endometrial tissues had a significantly greater survival rate at 10 weeks in SCID mice when compared to athymic nude mice. However, all SCID strains exhibit an increased susceptibility to opportunistic infections (both viral and bacterial) when compared to nude mice (Table 1). One of the most commonly used SCID strains is the non-obese diabetic SCID (NOD-SCID). NOD-SCID gamma (NOD.Cg-Prkdc^*scid*^ ll2rgtm1Wjl/SzJ or, simply, NSG) mice are also used. This strain was developed by Jackson Laboratory and is one of the most severely immunodeficient mice available (Pearson *et al.*, 2008). In addition to lacking mature T cells and B cells, NSG mice lack NK cells and have defects in innate immunity as a result of mutation in interleukin-2 receptor gamma (Il2rg), which is also known as the common cytokine receptor γ chain (γc) (Shultz *et al.*, 1995).

##### Rag2γ(c) mice

Recombinant activating gene 2/common cytokine receptor γ chain (γc) double null mice (rag2γ(c)) have a more global immunodeficiency affecting a broader group of immune cells than nude or many SCID mice. These mice were developed when Mazurier *et al.* (1999) crossbred the common gamma c knockout mouse (developed by (Cao *et al.*, 1993)) with recombinase-activating gene-2 (Rag2)-deficient mice (developed by (Alt *et al.*, 1992)). The resulting Rag2γ(c) strain demonstrated an absence of B cells, T cells, NK cell activity, and all lymphocytes (Table 1). Importantly, these animals do not display age-related compensatory immunity and, thus, can be used in studies of extended duration. Greenberg and Slayden first reported xenotransplantation of endometrial tissues into Rag2γ(c) mice, which were subjected to repeated cycles of oestradiol followed by oestradiol plus progesterone to induce four 28-day artificial menstrual cycles (Greenberg and Slayden, 2004). Mice were euthanized after the fourth cycle, and the majority were found to exhibit endometriotic-like disease.

The commercial availability of Rag2γ(c) mice is currently restricted to a single company (Taconic Biosciences, Rensselear, NY, USA). Although propagation of a colony is straightforward since females do not exhibit the lactation issues seen in nude mice, Taconic limits propagation to researchers with special permission.

##### Additional immunocompromized strains

In addition to nude, congenic SCID, and Rag2γ(c) mice, numerous other immunocompromized or immunodeficient strains have developed over the last several decades (reviewed in Mian *et al.* (2020) and Yang (2021)). In particular, back-crossing SCID mice onto strains with specific mutations led to the development of NOD-SCID gamma mice (described above and also known as NSG mice) and the less severely immunocompromized alternative Fox Chase SCID Biege (*Prkdc^scid/scid^* Lyst^bg/bg^) strain. These and other immunocompromized mouse strains have found favour with researchers studying the role of inflammation in cancer and other diseases and may have utility for endometriosis (Mian *et al.*, 2020; Yang, 2021). As before, the choice of the mouse strain to be used is primarily dependent upon the research question being addressed. Investigators should carefully weigh the pros and cons of each mouse strain alongside the hypothesis to be tested in order to find the best fit for their study.

#### Type of human tissues

The capacity to examine human endometrial and endometriotic tissues growing *in vivo* makes xenograft models of experimental endometriosis useful for a wide variety of studies. Indeed, such murine models have been used in many fields to determine the natural course of disease and to explore experimental conditions that impact its progression. For the study of endometriosis, human tissues or cells can be obtained from women with and without disease, thereby enabling investigators to compare the resulting phenotypes of experimental lesions. These models also lend themselves to identifying tissue differences that may impact ectopic survival, providing clues regarding not only disease establishment but also its progression.

Thoughtful human donor inclusion and exclusion criteria must be defined based on study goals and are described in detail in EPHect-EM-Heterologous SOP3 (Supplementary File S1). Briefly, for patients with endometriosis, recognizing the broad heterogeneity of phenotypic presentation among patients is critical (Horne and Missmer, 2022). Characteristics of human tissue donors to be defined, at a minimum, are exogenous hormone exposure status and menstrual cycle phase at tissue collection. Ideally, standard donor characteristics defined should also include endometriotic lesion macro-phenotypic presentation (superficial peritoneal lesions, any endometrioma, any deep lesions), non-hormonal medication exposure status (especially anti-inflammatory analgesics), age, parity, history of infertility, moderate or severe dysmenorrhoea and/or non-menstrual pelvic pain, uterine fibroids, or adenomyosis. For those without endometriosis but with non-endometriosis morbidities (e.g. uterine fibroids, chronic pelvic pain, infertility, autoimmune conditions), documenting and justification for inclusion or exclusion is critical (Missmer, 2019; Szklo and Nieto, 2019). Without a clear description of the criteria used for ‘healthy’ participants, causal inference cannot occur.

While some of these characteristics can be abstracted from the medical record in the detail required, most endometriosis phenotypic information is not documented adequately, if at all (Shafrir *et al.*, 2021). At a minimum, required characteristics can be orally or electronically collected about the human donor using a brief tissue collection-specific intake form. Ideally, comprehensive clinical metadata can be collected by completion of the WERF EPHect Patient Questionnaire (EPQ) (Vitonis *et al.*, 2014). For endometriosis macro-phenotypic details, the WERF EPHect Surgical Form (SSF) (Becker *et al.*, 2014) should be completed by the surgical staff collecting the tissue whenever possible as part of the standard protocol for heterologous tissue source models. If it is truly impossible to collect these data within the scope of the heterologous model study, absence of this knowledge should be documented, and that opacity should be considered in the inference of results.

When the number of human donors will be too small due to cost or feasibility to meaningfully explore heterogeneity among patient characteristics, then intentional *a priori* restriction to a single class of human donor (chosen specifically to maximize the validity of the experiment being conducted) should be clearly defined (e.g. late proliferative phase (Days 8–12), no hormone exposure, at least one endometrioma, no history of infertility, etc.). When restrictions are applied or when human donor phenotypic variation cannot be accounted for, the impact on generalizability of the experimental results to all endometriosis physiology or response must be considered, e.g. results may differ between heterologous models utilizing tissue collected only during the late proliferative menstrual cycle phase compared to models utilizing tissue collected from any phase of the menstrual cycle.

For establishment of experimental endometriosis, eutopic endometrium can be obtained by a qualified gynaecologist via eutopic endometrial biopsy during hysteroscopic surgery or in an outpatient setting using a plastic Pipelle de Cornier^®^ device. Experimental disease in mice has also been successfully induced using human menstrual effluent (Nisolle *et al.*, 2007). Several laboratories have also successfully established experimental disease using ectopic lesions obtained during surgery. However, the tissue take rate of experimental disease is variable, and resulting lesions are frequently contaminated by extraneous tissues (Bruner-Tran *et al.*, 2002).

Experimental disease has also been successfully established in immunocompromized mice using immortalized endometrial cell lines embedded in a matrix (Banu *et al.*, 2009). Although such studies, to our knowledge, have not been widely conducted, future studies utilizing heterologous models of endometriosis should benefit from ongoing attempts to establish multicellular endometrial organoids (also known as assembloids). At present, ‘endometrial’ organoids consist only of epithelial cells within a well-defined matrix (as described in our companion paper (Marr *et al.*, 2025)). Continued development of these systems is expected to lead to a multicellular organoid that more closely replicates the endometrium. Ideally, a complete endometrial organoid would consist of not only stromal and epithelial cells but also immune cells. Mixing of different populations of cells from healthy versus endometriosis patients or a variation of cell ratios within the matrix can be envisioned. Such combination studies would be invaluable for determining the contribution of each cell type to lesion establishment, progression, and/or response to treatment.

Human tissue collection requires review and approval by a human ethics committee. Consent must be informed, with participants given time to consider transparent information about the research, the time commitment, costs, remuneration, and any risks involved (infection, bleeding, and the unlikely possibility of damage to the uterine muscle). Inclusion and exclusion criteria should state any restrictions on medication use, ability to undergo informed consent (e.g. language or need for parental consent and patient assent for minors), and any requirement to reveal known medical conditions. A complete list of recommended inclusion/exclusion criteria is provided in EPHect-EM-Heterologous SOP3 (Supplementary File S1). Further criteria may be added to support the needs of a specific study. Additional considerations for specific tissue types are presented below.

##### Eutopic endometrium from women with and without endometriosis

For studies that will compare differences in the capacity of endometrial tissues from women with and without endometriosis, unfortunately, obtaining appropriate control human tissues from healthy women represents a major challenge. The ideal control tissue donor is a healthy woman exhibiting regular menstrual cycles and who has been surgically confirmed to not have endometriosis. Healthy, symptom-free women do not routinely undergo surgery unless they have a tubal ligation, which is increasingly rare, so recruiting such an ideal control donor is difficult. Women without symptoms or known risks of endometriosis (e.g. no severe menstrual pain, history of infertility, or first-degree relative with endometriosis) and with regular, natural, menstrual cycles are the next best choice for recruitment as a control donor. However, without visualizing the pelvis, there will always be some uncertainty whether a donor has endometriosis or not. Most often, the collection of control samples is obtained by pipelle biopsy in the outpatient clinic. Although not ideal, ‘control’ endometrial tissues have also been obtained from patients undergoing surgery for other gynaecological conditions (e.g. fibroids, benign cysts, or cervical conditions). For any study, it is critically important for all resulting publications to accurately describe the characteristics of their control population.

For donors with symptoms highly suggestive of endometriosis, eutopic tissue can be collected at laparoscopy to confirm the disease after informed consent. For women with ultrasound or MRI findings consistent with the presence of ectopic lesions, an endometrial biopsy can be performed during the initial therapeutic surgery, provided the patient has given informed consent. Although those with a history of surgical or medical treatment of endometriosis can be considered for an outpatient endometrial biopsy, we recommend against using these patients if the goal of the experimental study is disease development or response to therapy in comparison to disease or response of experimental lesions established by control tissues. Medical or surgical treatment of endometriosis leads to uncertainty regarding their current disease status. Additionally, partially treated disease or co-existence of adhesions likely alters endometrial tissue function, which will complicate the interpretation of studies.

Ideally, all tissue donors should have regular ovulatory cycles and must not be taking oral contraceptives or medications that disrupt menstrual cyclicity (EPHect-EM-Heterologous SOP3: Supplementary File S1). It is important to record endometriosis status, regularity of cycles, stage of menstrual cycle, medication use, and any other gynaecological conditions present at the time of tissue collection (see EPHect-EM-Heterologous SOP1b: Supplementary File S1). Care must be taken to ensure the amount of tissue injected per mouse is uniform within and between experiments (see EPHect-EM-Heterologous SOP7: Supplementary File S1 for complete details and recommendations).

##### Ectopic tissues

Endometriotic lesions occur in at least three main variants: peritoneal disease, deep endometriosis, and ovarian endometriomas (Bulun, 2009; Pasalic *et al.*, 2023). These lesions vary widely in the presence of endometrial stromal and epithelial cell ratios as well as in collagen and/or immune cell content. Xenografting endometriotic lesions into immunocompromized mice can aid in determining the impact of lesion phenotype in the establishment and progression of disease. However, of these variations, endometriomas best lend themselves to the establishment of experimental disease due to their consistently robust stromal and epithelial components (Hoyle and Puckett, 2024). The addition of human immune cells into such animals provides insight into how the lesion or tissue phenotype impacts immune response to disease.

##### Menstrual effluent

Experimental endometriosis has been successfully established following the introduction of tissues collected from human menstrual effluent. These samples can be obtained from the pelvis during surgery (Nisolle *et al.*, 2007; Gonzalez-Ramos *et al.*, 2008) or from the cervix via a menstrual cup (Koks *et al.*, 2000). Antegradely shed menstrual tissues express a variety of matrix-degrading enzymes (MMPs) and their inhibitors (TIMPs) for up to 24 h after collection. Several studies have suggested these enzymes may contribute to ectopic tissue engraftment (Bruner-Tran *et al.*, 2002; Ke *et al.*, 2021). Menstrual tissues from women with endometriosis vary in the presence of immune cells as well as in the expression of pro-inflammatory cytokines. Comparing menstrual samples obtained from women with and without endometriosis may provide important insight into the reasons why only a subset of women develop disease, despite the majority experiencing retrograde menstruation (reviewed in Herington *et al.* (2011a)). Menstrual tissues contain a higher proportion of blood and mucous compared to an endometrial biopsy. Regardless of the source, it is important to remove residual blood by repeated rinsing with PBS prior to introduction into mice to prevent induction of adhesions or, in the worst case, a potentially fatal cytokine-mediated immune response. Necrotic tissues in menstrual fluid are challenging to remove; however, they may provoke an innate immune response that enhances lesion formation.

##### Isolated endometrial cells

Primary and/or immortalized human endometrial cell lines embedded in a matrix have also been introduced into immunocompromized mice. Banu *et al.* (2009) created xenografts using immortalized human endometriosis epithelial cells and stromal cells (commonly, 12Z and B22 cells, respectively) embedded in Matrigel, which were injected into the peritoneal cavity of nude mice. The resulting lesions were found to attach, invade, reorganize, and proliferate. Similar lesions were also successfully created using isolated primary human endometrial stromal and epithelial cells resuspended in a hydrogel matrix. These artificial lesions reorganize and, at necropsy, mimic the histomorphology of the human disease (Fig. 2G–I). However, it is currently unknown whether and when these structures recapitulate the functionality of spontaneous peritoneal endometriosis in women. Indeed, the main criticism of this approach is that established cells lines used as donor tissue (commonly 12Z and B22) are exposed to gene deregulation during the *in vitro* culture, which likely alters the *in vivo* response. The isolation protocol used to obtain purified cultures of endometrial stromal and epithelial cells may also impact cell behaviour.

#### Hormonal status of tissue donor

It is essential to consider the cycle phase of the patient at the time of tissue collection. Many investigators prefer to acquire samples during the oestrogen-dominant proliferative phase (e.g. cycle days 8–12) since endometriosis is dependent on oestrogen for growth. Nevertheless, experimental disease has also been successfully established using tissues obtained during the secretory and menstrual phases of the cycle. Regardless of when the tissue is acquired, it is imperative to obtain and report this information.

The date of the patient’s last menstrual period (LMP) is a reasonable estimate of cycle phase but should ideally be confirmed with serum oestradiol (E2) and progesterone (P4) levels. A serum level of more than 300 ng/ml E2 and a P4 of <1.5 ng/ml is consistent with the late proliferative phase, whereas a P4 over 5 ng/ml indicates ovulation has occurred and dates the endometrial tissue in the mid to late secretory phase (Anckaert *et al.*, 2021). Paraffin-embedded histological sections and haematoxylin and eosin (H&E) staining can also be used to date the endometrium using Noyes criteria if some tissue is placed in formalin at the time of tissue collection (Noyes and Haman, 1953; Noyes *et al.*, 1975). Although dating this tissue by histology is always recommended, this process is unlikely to be completed prior to introduction into mice. Thus, most investigators rely on the patient’s reporting of her LMP. Dating from LMP is adequate if the donor is confident in the timing and her cycles are consistent. Cycle phase estimation can also be enhanced by having the participant return a prompted text message or email or prepaid postcard reporting the date of the first day of their next menses after the sample collection.

#### Preparation of human tissues

At the time of tissue collection, human endometrial samples (eutopic or ectopic) require placement in a sterile physiological media such as PBS, Ringer’s solution, normal saline, or pre-prepared tissue culture media. These solutions can be made available to the gynaecologist prior to the time of tissue collection. To ensure the survival of the biopsy, tissues should be refrigerated or placed on wet ice until use (Fassbender *et al.*, 2014). Although use of tissues should not be unnecessarily delayed, experimental endometriosis has been successfully established after overnight culture (Bruner-Tran *et al.*, 2002, 2006) or overnight transportation on wet ice (K. Bruner-Tran, personal communication). Prior to introduction into mice, eutopic or ectopic human endometrial biopsies must be washed to remove excess blood and mucous. Additional details may be found in the WERF EPHect collection tool for clinical data (Fassbender *et al.*, 2014) as well as in EPHect-EM-Heterologous SOP3 (Supplementary File S1).

##### *In vitro* culture prior to injection (optional)

Numerous studies have included *in vitro* treatment of human endometrial tissues with different factors (e.g. pharmaceuticals, steroids, inflammatory cytokines, seminal fluid, etc.) prior to injection into mice (Bruner-Tran *et al.*, 2002, 2006). If such an approach is desired, minced endometrial tissue can be maintained up to 24 h in culture at 37°C in a CO_2_ incubator. Spent media can be collected and analysed for secreted proteins. Prior to introduction into mice, tissues must be washed in sterile PBS in order to remove media and other additives.

##### Labelling for non-invasive tracking of lesions (optional)

(See EPHect-EM-Heterologous SOP4: Supplementary File S1.) In order to enhance identification of lesions at necropsy, enable non-invasive assessment of lesion size, or monitor disease development in live mice, investigators have developed methods to label human endometrial tissues with fluorescent tags prior to injection (Fortin *et al.*, 2003). However, it can be difficult to ensure adequate uptake of a fluorescent label in endometrial tissue fragments. For example, lentiviral-associated fluorescent/luminescent particles are not suitable as they have low tissue penetrance. To overcome this limitation, adenoviral vectors can be employed; however, they do not integrate within the genome and exhibit transient expression that fades over time. For this reason, lesion sizes estimated non-invasively using adenoviral fluorescent labels are not accurate beyond 30 days post tissue implantation (Gakhar *et al.*, 2009; Ferrero *et al.*, 2017), although identification of lesions post mortem is still greatly enhanced (Delgado-Rosas *et al.*, 2011).

In contrast to whole tissue fragments, isolated cells readily take up lentiviral-associated fluorescent/luminescent tags. This approach is compatible with assessing the initial stages of lesion attachment, lesion growth, and identification of lesions at necropsy.

#### Tissue placement and method

##### Intraperitoneal placement

*Injection*: (See EPHect-EM-Heterologous-SOP7: Supplementary File S1.) The majority of published studies have used intraperitoneal injection using an 18-gauge needle to transfer endometrial tissues suspended in PBS into mice. This method best mimics retrograde menstruation, requires no special equipment, and does not require analgesia of animals. However, anaesthesia of mice is important to reduce animal discomfort during the injection and to reduce the risk of injury to the investigator.

Despite the ease of this method for disease establishment, identification of unlabelled lesions at necropsy can be difficult since tissues can attach anywhere within the peritoneal cavity. Additionally, excision of lesions without surrounding mouse tissue can be challenging and can impair identification and quantification of genes and protein expression unless human-specific antibodies or primers can be used.

*Suture*: (See EPHect-EM-Heterologous-SOP8; Supplementary File S1.) Some investigators have opted to suture human tissues to specific locations within the peritoneal cavity. This method can be advantageous since the investigator knows the number of lesions that were placed as well as their location. Tissues can also be purposefully located near a blood supply to facilitate survival and growth. However, surgical induction of disease is a major abdominal surgery and requires not only a skilled surgeon, but both anaesthesia and post-surgical analgesia. Inflammation associated with surgery may impact some endpoints (e.g. ovarian, uterine function, immune environment of the peritoneal cavity, pain-like behaviour) and thus sham animals, which undergo laparotomy but are not induced with disease, are an important control group that must be included. Alternatively, several investigators have reported the successful use of fibrin glue to establish experimental endometriosis in rats using homologous tissues (Boztosun *et al.*, 2012). It is likely this method would also be amenable to heterologous mouse models of experimental human endometriosis.

##### Subcutaneous placement

*Injection*: (See EPHect-EM-Heterologous-SOP7: Supplementary File S1.) Like intraperitoneal injection (IP), subcutaneous (sc) injection of tissues is relatively easy and does not require exceptional skill or analgesia of mice. Although this approach is less representative of human ectopic lesions, which are predominantly exposed to the peritoneal environment, humans have also been found to develop subcutaneous endometriosis, most commonly in Caesarian scars (Zhang *et al.*, 2019). For this approach, an 18-gauge needle is used to transfer minced human endometrial tissue, which is placed within the subcutaneous layer overlying the anterior peritoneum ventrally, usually along the midline of the mouse. Subcutaneous lesions are readily identifiable at necropsy, reducing accidental excision of non-human tissues. The confined subcutaneous tissue compartment promotes engraftment, and the size and weight of lesions more closely correlate with the injected tissue load when compared to tissues injected into the peritoneal cavity, where some fragments can fail to establish as a lesion. Additionally, subcutaneous injection of tissues into nude mice enables external visualization of larger lesions and non-invasive monitoring using callipers (Fig. 3A).

##### Kidney capsule

At least two groups have reported the establishment of endometrial tissues placed under the kidney capsule (Kurita *et al.*, 2005; Bruner-Tran *et al.*, 2018). Advantages of this method include readily identifiable lesions compared to those developing after intraperitoneal injection of tissues and the vascularity of the kidney, which may enhance survival of ectopic tissues. Additionally, the kidney capsule can be used for the insertion of whole tissue explants as well as isolated cells that have be recombined within a matrix as describe above. This method enables the exploration of different combinations of endometrial cells, potentially allowing more precise determination of cells, which may promote disease. However, this method requires major abdominal surgery and is technically challenging.

#### Hormonal status of recipient animal

##### Endogenous versus exogenous hormones

Oestrogen causes proliferation of endometrial tissues and thus plays a critical role in the establishment and growth of ectopic endometrial-like lesions (Bulun *et al.*, 2012). In contrast, progesterone or progestogens attenuate disease development (Bulun, 2009; Bulun *et al.*, 2012) and are used therapeutically in women to moderate pain symptoms and endometriotic disease progression. However, a majority of women with endometriosis exhibit a reduced responsiveness to progesterone; thus, progestin-based therapies are not effective for all patients (Bruner-Tran *et al.*, 2002; Clemenza *et al.*, 2023; Hon *et al.*, 2023; Zhang and Wang, 2023).

Progesterone resistance resulting from dysregulation of aromatase and COX2 pathways in eutopic endometrial cells has been reported in women with endometriosis and is purported to negatively impact embryo implantation and/or facilitate peritoneal engraftment of shed cells in retrograde menstrual fluid (Giudice *et al.*, 2002; Kao *et al.*, 2003). Progesterone action in women is largely mediated via progesterone receptor-B (PGR-B), while PGR-A, a truncated isoform of PGR-B, can act as a dominant repressor (Vegeto *et al.*, 1990, 1993). Several studies have reported reduced endometrial expression of PGR-B and increased expression of PGR-A in patients with endometriosis compared to healthy women (Attia *et al.*, 2000; Igarashi *et al.*, 2005; Wolfler *et al.*, 2016). Oestrogen also acts via specific receptors, oestrogen receptor-α (ESR1) and oestrogen receptor-β (ESR2), the latter of which has been demonstrated to be overexpressed in women with endometriosis and may play a role in disease development (Bulun *et al.*, 2012; Simmen and Kelley, 2016; Stocks *et al.*, 2017).

Since reproductive steroids contribute significantly to the development and progression of endometriosis, an important consideration in heterologous experiments is whether or not to leave the mice intact or remove the ovaries. Ovariectomized mice can be induced to exhibit an artificial cycle that more closely mimics the human or provided continuous oestrogen in order to promote more robust disease. This decision is left to the individual investigator depending on their needs; however, herein, we provide information for consideration.

##### Intact mice

For studies conducting preclinical drug testing, the use of intact mice is often preferred. Although the murine oestrus cycle does not fully replicate the cellular responses associated with the menstrual cycle in engrafted tissues, it is useful to examine potential treatment effects on the murine ovary and/or uterus (Arosh *et al.*, 2015; Palmer *et al.*, 2016). While it may be theoretically possible to conduct studies examining the impact of experimental endometriosis on fertility, this line of investigation is not recommended within a xenograft model due to the immunocompromized state of the host. For such studies, it is our recommendation that the homologous mouse model of experimental endometriosis be used (Burns *et al*., 2025).

If intact animals are used for experimental endometriosis, all animals must be euthanized during the same stage of the oestrous cycle for normalization of data. Oestrous staging of mice can be conducted by visual assessment of the vaginal opening (EPHect-EM-Heterologous-SOP5: Supplementary File S1) or by microscopic assessment of cells following a vaginal swab (Zenclussen *et al.*, 2014).

##### Ovariectomy plus oestrogen only supplementation

Ovariectomized animals supplemented with oestradiol capsules or pellets may be preferred for studies focused primarily on mechanisms associated with lesion development, as this regimen more fully represents the human proliferative phase. Oestrogen (E2) delivery is simplified using subcutaneously implanted commercially available steroid pellets (e.g. Innovative Research of America) or can be made in-house (EPHect-EM-Heterologous-SOP6a: Supplementary File S1). Given the E2-dependent progression of endometriosis, continuous administration of E2 is often used to maximize lesion growth and survival by promoting proliferation and angiogenesis (Bruner-Tran *et al.*, 2010). Nevertheless, not all studies have found that the growth and implantation of lesions is enhanced by oestradiol (Grummer *et al.*, 2001; Colette *et al.*, 2009).

Exogenous administration of oestrogen can also have off-target and/or confounding effects in murine models. Supraphysiological oestrogen dosing can lead to systemic changes such as uterine growth (Edwards *et al*., 2013), cell proliferation (Groothuis *et al.*, 2007), reduced NK cell activity (Seaman and Gindhart, 1979), or alteration of miRNA profiles (Nothnick and Healy, 2010), among other effects. Additionally, ∼50% of nude mice will develop urinary retention with uni- or bi-lateral hydronephrosis when exposed to supraphysiologic oestrogen for more than three weeks (Gakhar *et al.*, 2009).

##### Ovariectomy and artificial cycles

The impact of menstrual cyclicity on the effectiveness of a potential pharmaceutical agent can be investigated in animals subjected to ovariectomy and artificially cycled. Menstrual cyclicity can be simulated in ovariectomized mice supplemented with oestrogen followed by oestrogen plus progesterone. As above, steroids can be provided using subcutaneously implanted steroid pellets or capsules. Although some investigators have successfully induced human menstrual-like cyclicity using daily injections of steroids (reviewed by Liu *et al.* (2020)), this method is labour-intensive and introduces stress as an additional variable.

#### Adoptive transfer of human immune cells

(See EPHect-EM-Heterologous-SOP9: Supplementary File S1.) Although the phenotype of endometrial tissue fragments significantly contributes to disease pathogenesis, the immune system also plays a critical role in disease development. For this reason, endometriosis is now considered a systemic inflammatory disease (Taylor *et al.*, 2021). Menstrual fluid from retrograde menstruation triggers an influx of immune cells into the peritoneal cavity, which normally clear any dead and dying endometrial cells present. When the engraftment and growth of the endometrial tissues outpace the ability of the immune cells to scavenge refluxed endometrial tissues, a lesion develops. Inflammatory mediators produced by both the immune cells and menstrual debris likely aid both the invasive capacity of tissues and neoangiogenic processes necessary for the establishment of ectopic lesions (Nothnick and Healy, 2010; Herington *et al.*, 2011a). Investigators can adoptively transfer human immune cells into the circulation of SCID, NOD-SCID, or Rag2γ(c) mice to examine the potential role of the immune system or specific immune cells in promoting or preventing the development of endometriosis. EPHect-EM-Heterologous-SOP9a (Supplementary File S1) describes isolating peripheral blood mononuclear cells (PBMCs) and polymorphonuclear leukocytes (PMNs; also known as neutrophils) from peripheral blood, while EPHect-EM-Heterologous-SOP9b (Supplementary File S1) describes fluorescently tagging these cells.

#### Outcome measures and endpoint assessments

At a minimum, mouse models of endometriosis require assessment of lesion size, number, location, and gross and microscopic appearance. Additional endpoints (e.g. response to treatment, impact of environmental factors on disease, effect of disease on other processes) are dependent upon the research question(s) asked and the type of experimental manipulation undertaken. Here, we provide recommendations and SOPs (Supplementary File S1) for collection of data at necropsy, microscopic examination of lesions, and documentation of data.

##### Examination of lesions

(See EPHect-EM-Heterologous-SOP10: Supplementary File S1.) The gross appearance of lesions can vary widely, depending on location and vascularization (Fig. 3). Before euthanasia, the condition of the animal should be assessed and any abnormalities that indicate pain or distress (e.g. vocalization, hunched posture) or poor health (e.g. piloerection of hair) or reduction in weight should be noted. Animals with oestrus or artificial cycles should be euthanized at the same cycle stage to reduce reproductive hormone variability, and the postmortem uterus should be weighed and photographed.

Euthanasia standards are prescribed by each institute or government animal care and use committee. Most investigators euthanize rodents with CO_2_ followed by another method to confirm mortality, such as aortic resection or cervical dislocation.

At post-mortem, a careful assessment of the subcutaneous space, peritoneum, and peritoneal cavity should be conducted. Lesions should be photographed and measured in at least two dimensions using a ruler or callipers. The location, size, colour, and extent of vascularization of each lesion should also be recorded. Consideration should be given to recording: individual and total lesion weight (if lesions are too small to weigh separately, total lesion weight is acceptable), presence and location of adhesions, presence of swollen lymph nodes, enlarged spleen, and/or abnormal appearance of other organs.

It is important to work quickly and keep excised tissues on wet ice until they have been weighed, measured, and documented. For nucleic acid assessment, lesions should be snap frozen in liquid nitrogen or stored in RNA later (Fassbender *et al.*, 2014). Fixing lesions in formalin or paraformaldehyde before sectioning for histological or immunohistochemical analysis permits qualitative and quantitative assessment of glands, stroma, haemosiderin macrophages, and fibrosis. Microscopic lesions should be examined by a skilled gynaecologic pathologist and/or endometriosis research scientist for confirmation by histology.

Different experimental designs may include the collection of other organs with uteri, ovaries, blood, and peritoneal lavage being commonly obtained. Additional samples, such as liver, spleen, intestine, spinal cord, and brain, may also have value. We recommend collecting as many tissues as possible for potential future analysis. Collaborations to determine the impact of experimental endometriosis on another organ system can then be explored at a later date.

There are several methods to determine the murine or human origin of cells in xenografts from the heterologous mouse model. Species-specific antibodies such as anti-human von Willebrand’s factor (which stains human but not mouse endothelial cells), the use of mice with fluorescently tagged cell lineages (for example, MacGreen mice bred onto an SCID background demonstrate fluorescent green macrophage staining (Johan *et al.*, 2019)), and the use of Hoechst histochemical counterstaining (stains human nuclei homogenously but murine nuclei with a punctate pattern (Hull *et al.*, 2003, 2008)) are all histochemical methods to determine the species origin of cells in mixed species tissues. Use of methods designed to identify species-specific orthologs enables investigators to obtain species-specific probes for PCR and *in situ* hybridization (Hull *et al.*, 2008).

##### Assessment of ‘pain’ endpoints

Evaluation of lesion dynamics can provide a wealth of information on mechanisms of lesion development and progression and, where appropriate, treatment efficacy on lesion survival (Martinez *et al.*, 2019). Additionally, assessment of clinically relevant endpoints can provide a better understanding of endometriosis symptomatology as well as treatment efficacy and, in doing so, further validate experimental rodent models of endometriosis. Pelvic pain is one of the main complaints associated with endometriosis in women, and its resolution is viewed as successful treatment in such patients.

Pain perception cannot be verbalized by rodents, necessitating the establishment of surrogate measures of pain. As described extensively in our companion paper (Dodds *et al.*, 2025), current methodology allows for assessment of both reflexive (evoked) and spontaneous (non-evoked) pain-like behaviours. Although to date, most pain models of experimental endometriosis have utilized homologous models, Gomez and colleagues recently conducted similar assessments of pain in mice bearing human tissue lesions (Tejada *et al.*, 2022, 2023). Methods for assessing evoked pain include Hargreaves/thermal hyperalgesia testing, visceromotor reflex, filament (von Frey) testing, and escape response to vaginal distention (Tejada *et al.*, 2022; Dodds *et al.*, 2025). Methods for assessing non-evoked or spontaneous pain include abdominal squashing, contortions and licking, and dynamic weight bearing (Tejada *et al.*, 2023; Dodds *et al.*, 2025). Additionally, ethological behaviour assessment (facial grimace) and assays related to thigmotaxis (open field activity, elevated plus/zero maze, burrowing, nest building, and home cage analysis) have also been utilized (Tejada *et al.*, 2023; Dodds *et al.*, 2025). Assessment of both evoked and non-evoked pain can be conducted at single or multiple time points during an experiment and can be tailored to fit the experimental design and hypothesis being tested. SOPs for these and other pain assessment methods are available in conjunction with our companion paper (Dodds *et al.*, 2025).

#### Replicates

It is difficult to recommend a specific number of replicates for each experiment due to patient variability and the unpredictable nature of biopsy sample size. Additionally, heterologous models require that two types of replicates be considered: the number of human samples used and the number of mice per group. Power calculations can be conducted prospectively based on estimates of the anticipated number of human donors and an average number of mice, which are expected to be utilized. However, investigators should be prepared to increase the sample sizes if estimates are not accurate. Assistance from a statistician is recommended to appropriately determine power calculations and to assess significance.

Pipelle de Cornier^®^ biopsies of eutopic endometrium conducted by an experienced gynaecologist will often provide sufficient tissue for eight or more mice, provided the endometrial thickness is robust. Setting aside a portion of endometrium for fixation and freezing is always recommended, though this will reduce tissue availability for establishing experimental disease. Overnight culture also reduces endometrial tissue mass. We recommend a minimum number of three different donor endometrial tissues when using control (endometriosis-free) human endometrial samples. Due to the wide range of disease presentations, we recommend a minimum of five different samples from those with endometriosis. As the number of mice that can receive implanted endometrial tissue from a single sample varies widely, more human samples may be required to ensure an adequate number of animal replicates.

Ideally, a single patient’s sample should be distributed across all mouse treatment groups. A minimum of two mice per group is also recommended. Following these recommendations, four is generally the maximal number of treatments able to be examined in a single experiment, although if menstrual tissue is used, this is limited to two. If large numbers of murine groups are required (for example, dose response experiments), a practical approach is to compare only two doses with an untreated control group across a series of studies.

## Discussion

More than a century since endometriosis was first described in the literature, we still do not understand its cause, nor have we identified an effective and tolerable long-term treatment. Nevertheless, the use of experimental rodent models has significantly broadened our understanding of endometriosis. For example, studies using rodent models of endometriosis have aided in identifying mechanisms associated with early lesion establishment, development of associated diseases (e.g. adenomyosis and adhesions), as well as the impact of disease on fertility, pregnancy, and offspring health (Stilley *et al.*, 2009; Herington *et al.*, 2011b; Bruner-Tran *et al.*, 2016; Kanellopoulos *et al.*, 2022; Kalehoei *et al.*, 2023). Rodent models of endometriosis have also been invaluable in assessing the effectiveness of potential therapies (Bruner-Tran *et al.*, 2009; Delgado-Rosas *et al.*, 2011; Amaya *et al.*, 2014; Ahmad *et al.*, 2015; Palmer *et al.*, 2016; Bruner-Tran *et al.*, 2018; Koch *et al.*, 2018; Ermiş *et al.*, 2023).

Herein, we have focused specifically on studies using heterologous mouse models of experimental endometriosis. These models establish disease by introducing human endometrial tissues, endometriotic lesions, or isolated endometrial cells into immunocompromized mice. Our review of published studies identified numerous unnecessary variations in model design and endpoint assessments, and, often, a lack of transparency regarding critical aspects of the experimental design and/or the human samples utilized. These variations and lack of detail impede reproducibility between laboratories and limit the translation of data to the human condition.

Although it was not feasible to review every study, we attempted to provide an overview of studies that highlight various heterologous models of experimental human endometriosis. We discuss both the advantages and disadvantages of each model and have provided a list of considerations for study design. Although no single model can replicate all aspects of human endometriosis, each has the potential to produce robust and valuable data. For each investigator, the choice of model used should be determined by the research question to be addressed using a ‘best fit’ strategy. To aid investigators in determining the most appropriate model given their study design and access to human samples, we produced a decision tree (Fig. 1). We additionally produced 10 primary and 5 adjunct SOPs developed from protocols currently used in the laboratories of the working group (Supplementary File S1). Importantly, we recognized the need for specific guidance regarding record keeping of both study design and endpoint assessment of lesions at post-mortem. Thus, we developed a specific SOP (EPHect-EM-Heterologous-SOP1: Supplementary File S1) to address this need using a template previously produced by Parker *et al.* (2015).

Finally, based on the literature review and discussions with members of the EPHect experimental models heterologous working group, we can make the following recommendations.

1. Injection of human tissues into the peritoneal space of mice is preferred. However, subcutaneous injection or suturing of tissues may be appropriate for certain studies.
2. The number of mice within a group, but also the number of human samples utilized, must be considered when determining experimental replicates and power calculations used to inform sample size.
3. Hormonal status of the patient from which human samples are obtained must be verified and reported.
4. When possible and appropriate, preclinical studies of new therapies should be conducted in more than one model as no one model replicates endometriosis entirely.
5. At necropsy, multiple tissues and organs should be collected and fixed and/or frozen. Collection of these additional samples will be a valuable resource for future study and/or collaborations.
6. Publish (in Supplementary Materials) details of the election of the humans from whom tissue was collected (‘healthy’ women or ‘women with endometriosis’ is insufficient for inference and between-study comparisons), the model protocols, and endpoint quantifications.
7. Publish negative data. Determining that a therapeutic agent was ineffective is valuable information that will change the direction of the field and reduce experimental redundancy.

Widespread adoption of these recommendations and SOPs is expected to improve the comparison of data across laboratory groups. It will also aid collaboration between investigators and will reduce the replication of studies unlikely to advance the field. Finally, we anticipate that the adoption of these recommendations and protocols will reduce barriers for new researchers who would like to establish heterologous models of endometriosis within their own laboratory. Harmonization of methods and enabling and encouraging new investigators to study endometriosis should have a dramatic, positive impact on our understanding of endometriosis and will facilitate the development of new therapies, which will improve the lives of millions with endometriosis across the world.

## Supplementary Material

gaaf022_Supplementary_Data

## Data Availability

No new data were generated or analysed in support of this research.

## Appendix: EPHect experimental models working group

Nick A. Andrews: Salk Institute for Biological Sciences, San Diego, CA, USA

Michael S. Anglesio: University of British Columbia, Vancouver, BC, Canada

Caroline B. Appleyard: Ponce Health Sciences University-Ponce Research Institute, Ponce, PR, USA

Joe Arosh: Texas A&M University, College Station, TX, USA

Christian M. Becker: University of Oxford, Oxford, UK

Kaylon L. Bruner-Tran: Vanderbilt University Medical Center, Nashville, TN, USA; World Endometriosis Research Foundation, London, UK

Katherine A. Burns: University of Cincinnati College of Medicine, Cincinnati, OH, USA

Ronald L. Chandler; Michigan State University College of Human Medicine, Grand Rapids, MI, USA

Julie A. Christianson: University of Kansas Medical Center, Kansas City, MO, USA

Fiona L. Cousins: Hudson Institute, Clayton, VIC, Australia; Obstetrics and Gynaecology, Monash University, Clayton, VIC, Australia

Kelsi N. Dodds: Flinders University, Adelaide, SA, Australia; University of Adelaide, Adelaide, SA, Australia

Victor Fattori: Harvard University, Cambridge, MA, USA

Asgi Fazleabas: Michigan State University, Grand Rapids, MI, USA

Caroline Gargett: Hudson Institute of Medical Research, Clayton, VIC, Australia

Juan S. Gnecco: Tufts University, Medford, MA, USA

Raul Gomez; INCLIVA Instituto de Investigación Sanitaria, Valencia, Spain; University of Valencia, Valencia, Spain

Martin Götte: University of Münster, Münster, Germany

Erin Greaves: University of Warwick, Warwick, UK; World Endometriosis Research Foundation, London, UK

Linda G. Griffith: Massachusetts’s Institute of Technology, Cambridge, MA, USA

Patrick G. Groothuis: Byondis BV, Nijmegen, The Netherlands

Ruth Grümmer: University of Duisburg-Essen, Essen, Germany

Sun-Wei Guo: Fudan University, Shanghai, China

Shannon M. Hawkins: Indiana University School of Medicine, Indianapolis, IN, USA

M. Louise Hull: The Robinson Research Institute, University of Adelaide, Adelaide, SA, Australia

Lone Hummelshoj: World Endometriosis Research Foundation, London, UK

Mark R. Hutchinson: University of Adelaide, Adelaide, SA, Australia

Mohamed Gamal Ibrahim: Team Kinderwunsch Oldenburg GbR MVZ, Oldenburg, Germany

Elizabeth E. Marr: Tufts University, Medford, MA, USA

Stacy L. McAllister: Emory University School of Medicine, Atlanta, GA, USA

Stacey A. Missmer: Harvard TH Chan School of Public Health, Boston, MA, USA; University of Michigan, Ann Arbour, MI, USA; World Endometriosis Research Foundation, London, UK

Jeffrey S. Mogil: McGill University, Montreal, QC, Canada

Jens Nagel: Merz Therapeutics, Frankfurt, Germany

Warren B. Nothnick: Kansas University Medical Center, Kansas City, MO, USA

Paulina Nunez-Badinez: Bayer AG, Wuppertal, Germany (until 23 March 2023)

Kevin G. Osteen: Vanderbilt University Medical Center, Nashville, TN, USA

Daniëlle Peterse: Boston Childrens Hospital, Harvard Medical School, Boston, MA, USA

Michael S. Rogers: Boston Childrens Hospital, Harvard Medical School, Boston, MA, USA

Andrea Romano: Maastricht University, Maastricht, The Netherlands

Philippa T.K. Saunders: University of Edinburgh, Edinburgh, UK

Miguel Ángel Tejada: University of Granada, Granada, Spain

Kathy L. Sharpe-Timms: University of Missouri School of Medicine, Columbia, MO, USA

Waldiceu A. Verri: State University of Londrina, Londrina, Brazil

Paola Viganó: Fondazione IRCCS Ca’ Granda Ospedale Maggiore Policlinico, Milano, Italy

Katy Vincent: University of Oxford, Oxford, UK
