# Correction to: WERF Endometriosis Phenome and Biobanking Harmonisation Project for Experimental Models in Endometriosis Research (EPHect-EM-Heterologous): heterologous rodent models

**DOI:** 10.1093/molehr/gaaf033

**Published:** 2025-07-28

This is a correction to: M. Louise Hull, Raul Gomez, Warren B. Nothnick, Ruth Gruemmer, Katherine A. Burns, Mohammed Zahied Johan, Isabella R. Land, Stacey A. Missmer, Lone Hummelshoj, Erin Greaves, and Kaylon L. Bruner-Tran, for the EPHect Experimental Models Working Group, WERF Endometriosis Phenome and Biobanking Harmonisation Project for Experimental Models in Endometriosis Research (EPHect-EM-Heterologous): heterologous rodent models, *Molecular Human Reproduction* Volume 31, Issue 3, 2025; gaaf022, https://doi.org/10.1093/molehr/gaaf022.

In the originally published version of this article, the following sentence in the sub-section ‘Types of human tissues’ within the Results section required correction: ‘*Without a clear description of the criteria used for ‘healthy’ participants, causal inference can occur*.’ The sentence has been corrected to read as follows: ‘*Without a clear description of the criteria used for ‘healthy’ participants, causal inference cannot occur.*’

