# Supplementary material for: WERF Endometriosis Phenome and Biobanking Harmonisation Project for Experimental Models in Endometriosis Research (EPHect-EM-Heterologous): heterologous rodent models
Source: Mol Hum Reprod. 2025 Jul 9;31(3):gaaf022. doi: 10.1093/molehr/gaaf022 (PMC12237513; doi:10.1093/molehr/gaaf022)
Supplement: gaaf022_Supplementary_Data [file gaaf022_supplementary_data.zip › MHR-24-0371-R1-SuppFileS1.pdf]

M. Louise Hull, Raul Gomez, Warren B. Nothnick, Ruth Gruemmer, Katherine A. Burns, Mohammed Zahied Johan, Isabella R. Land, Stacey A. Missmer, Lone Hummelshoj, Erin Greaves, and Kaylon L. Bruner-Tran for the EPHect Experimental Models Working Group.

TABLE of CONTENTS

SOP 1: General experimental documentation 2
SOP 1a: Documentation for experimental endometriosis 6
SOP 1b: Participant identification and documentation 7
SOP 2: Universal precautions 9
SOP 3: Human endometrial tissue collection 10
SOP 4: Labelling of endometrial tissue 12
SOP 5: Visual assessment of the murine cycle stage 14
SOP 6: Ovariectomy and steroid capsule placement 15
SOP 6a: Preparation of silastic capsules with oestradiol 17
SOP 7: Experimental human endometriosis in immunocompromised mice: Injection method 19
SOP 8: Experimental human endometriosis in immunocompromised mice: Suture method 21
SOP 9: Retro-orbital injection of immune cells 23
SOP 9a: Isolation of PBMCs 24
SOP 9b: CFSE fluorescent labelling of PBMCs 26
SOP 10: Macroscopic assessment of lesions 28

SOP 1: General experimental documentation

Purpose
This procedure outlines the steps involved in proper recording keeping in the laboratory. Appropriate documentation of experimental research is critical for both interpreting and publication of research data. Not all sections will be relevant for all experiments; however, all studies require adequate documentation that will enable the reader to understand and replicate the study.

1.0 Definitions

Research Integrity: Intellectual honesty in the process of acquiring and extending knowledge; adherence to ethical requirements in their research; and public dissemination of research for peer review.

Responsible Conduct of Research: Conduct research that is open and honest; accurately disseminate study results; comply with applicable institutional policies, professional standards and applicable laws and regulations. Additionally, researchers are expected to keep complete and accurate records of data, methodology, and findings such that their work can be verified and/or replicated by others.

Individual laboratory notebook: A bound notebook, with consecutively numbered pages, kept in the laboratory at all times. Any researcher conducting experiments must include detailed information pertaining to each experiment conducted. Additional information, such as statistical analyses and interpretation of data, should be included if available. The level of detail recorded must be sufficient to allow the work to be reproduced, by others, without the original researcher. If a procedure is used repeatedly, a reference to the detailed protocol with version date) must be made each time it is performed. If a protocol is later modified (which requires it to be saved with a new version date), a copy of the version that was used for the experiment work must be kept.

Individuals working on multiple projects are encouraged to have a laboratory notebook dedicated to each project.

Task Notebooks: For certain studies, it may be helpful to have a dedicated notebook that multiple investigators use. For example, Necropsy notebook (or notebooks, if multiple studies are being conducted) is useful to keep in the laboratory in which animals are euthanized so that information can be recorded in real time.

Inventory binder or notebook: an inventory book used to record what supplies (including supplier, kit and lot numbers, etc.), samples and materials were used and/or generated by work in the laboratory and their location within the laboratory.

Electronic research records: primary and secondary data files used to record the output of experimental analyses from instruments (“source” or “primary” electronic datafiles) and to further analyze research findings.

2.0 Equipment/materials

- Laboratory notebook
- Laboratory inventory binder or notebook
- Laboratory computer for housing electronic data files Laboratory and personal USB/external hard drives

3.0 Action/Decision-making Framework

3.1 Maintaining/updating laboratory notebooks

Labeling laboratory notebook: The cover page of a laboratory notebook must contain the research members full name, the notebook number (sequential) and year (i.e. Book #1, 2022; Book #2 2023, etc.) clearly marked on the cover of each

laboratory notebook. The pages within the notebook must be consecutively numbered. It is recommended that a table of contents be recorded, either at the beginning or end of the notebook, to help organize and locate records.

Entering data into laboratory notebooks: On a daily basis, experimental plans and any data generated must be entered into the laboratory notebook, with the date that the plans were made and/or the experiment was conducted. The entry should include the location where any primary data files are stored (e.g. "raw data located on main computer by microplate reader; C:\Users\User\Documents\ nameofPI\ELISA\20140214.doc). If appropriate (e.g., in the case of multiple instruments in the facility), the make, model, serial number and location of equipment used for analysis should be noted in the records. Each member of the research team is expected to paste a copy of the raw data, the analyzed data (e.g. graphs) and any statistical analysis performed for experiments. Ideally, records should outline the rationale, hypotheses, all methodologies, results and conclusions. It is expected that research team members record all relevant details (e.g., which subjects' samples were tested, details of dilutions, volumes of materials tested per lane or well, etc), observations, interpretations and comments about single or multiple experiments in laboratory notebooks. This will facilitate repeating experiments and troubleshooting any problems that arise.

Notes

- Pens must be used to enter records in laboratory notebooks. The use of pencil or water-soluble ink is not acceptable.
- Removal of pages or "white-out" any content is not allowed. Errors or changes in plans may be indicated by crossed out the information with a single line through the erroneous text.
- Laboratory notebooks and inventory records must not be removed from the laboratory as primary research records must be maintained by laboratory for research integrity.
- If an experiment is performed with, or for another research team member, it is recommended that both individuals each keep a copy of the records in their notebooks.

Practices that are encouraged: Members of the research team are advised and encouraged to create, maintain and continuously update an electronic narrative of their work (as a draft thesis or paper) to help organize their thoughts and facilitate inclusion of sufficient details of their work. It should be organized as: background/introduction, materials and experimental methods, results and discussion. If a procedure is to be used repeatedly without variance in procedure, consider preparation of a standard operating procedure (SOP; with version date). Literature searches should be performed before undertaking work, and periodically during a project, to ensure that the research plans adequately consider current knowledge.

4.0 Saving files on laboratory computers

All primary (source) electronic data files, and secondary files (e.g., data analysis or graphs) generated for the experimental work must be saved on the computers in the laboratory (e.g. on the C-drive or network drive, not just on an external drive; never save primary data on USB devices except for transfer). All data must be logically organized into folders and must be clearly labeled (e.g., name of team member, date and type of experiment conducted). It is forbidden to delete or overwrite data files from the computer records held by the laboratory (see Section 7.4). Source data files (electronic and hardcopy records) must be kept for all experimental work. Files generated on computers outside the laboratory (e.g., from sequencing, real- time PCR files, collaborators instruments) must be copied over to a laboratory computer to ensure that records are complete. If work is done offsite, or if analyses are completed on a personal computer, a copy must be transferred back onto a laboratory computer to ensure the integrity and completeness of records. If data files from multiple experiments are used to create a graph, table or narrative, maintain a record of which experiments were used (by date and type of experiment), and any data for similar experiments that were excluded (e.g., if there were failed experiments, or pilot work to develop a protocol). Inventory records should list which laboratory

computers contain the source and secondary data files. An experiment that isn't properly documented is considered a failed experiment that can never be published or presented.

4.1 Creating back-up files of records on the lab computers

All files on the hard drives of laboratory computers will be back-up on a weekly basis by the senior laboratory personnel to ensure the integrity of record keeping. Access to the external hard-drives or network drives with experimental data files requires permission of both the senior laboratory personnel and the supervisor.

4.2 Removing data from the lab

Laboratory notebooks and other hard copy experimental records (including inventory records) can never be removed from the supervisor's laboratory. It is also forbidden to delete or overwrite electronic data files that document experimental work on the supervisor's laboratory computers. Members of the research team are encouraged to make photocopies of hard copy files and to create their own electronic backup files to further ensure the integrity of their research records (e.g. using USB or External Hard Drives to transfer files) and the timely presentation and publication of work.

4.3 Maintaining/updating a personal laboratory inventory

Any and all materials used to conduct laboratory work must be listed in inventory records. General project, or personal project binders (in the case of sole users), must be used to store information on product sheets. Ensure that all materials have an acceptable Material Safety Data Sheet (MSDS) in the common laboratory binder with MSDS records (a required safety resource of the laboratory). Use a sample inventory book to keep an organized record of primary and secondary samples and materials and the location where they are stored. If possible, also state the quantity of material remaining and the date this was recorded. This inventory must be updated regularly to ensure accurate and up-to-date information. The purpose of personal laboratory inventories (which do not replace the general laboratory inventory records) is to help members of the research team to locate any/all samples and materials used for laboratory work on a project. Make sure that these inventories are up-to-date when new samples or supplies are prepared/received, and ensure that all supplies used are listed on the laboratory reagent inventory and MSDS records.

4.4a Recordkeeping related to samples from human

Each university or other research entity has varying requirements for how human data is collected and stored. In general, only relevant medical information is obtained and subject identities are kept anonymous. All subjects that donate samples must be fully informed and provide written consent prior to collecting samples for research purposes. Typically, a unique, and anonymized, character code will be created and assigned to each subject used in research studies. Only anonymized codes can be used in laboratory records and on sample labels. Signed consent forms, and records that link the anonymized code to a subject are kept in a locked filing cabinet in the supervisor's office, in accordance with REB policies. It is forbidden to indicate name of a patient or control in laboratory notebooks, data files, inventories or publications. Sample labels should include the type of sample, and date of collection, if a donor is used more than once and/or to prepare more than one type of sample. An inventory of all patient and control samples must be maintained with the following details (not in this specific order): unique anonymized code; who prepared and collected the sample; type of sample; procedure used to collect samples; purpose of an experiment on a sample; and date and details of the experiment.

4.4b Record keeping for work done in collaboration with other laboratories

All data files generated in other research laboratories as part of collaborative work must be stored in a separate folder on the computers in the collaborating laboratory (in keeping with requirements of the collaborator), and an exact copy of the folder is to be maintained on a computer in the primary supervisor's laboratory. Files must be clearly labelled with the name of the laboratory. All data must be stored and updated as described in sections 7.2 and 7.3. Similarly, all experiments conducted in collaborating labs must be properly recorded in a laboratory notebook. If the collaborator requires that a laboratory notebook be kept in their laboratory for the collaborative experimental work, there must be

a complete replica of the record in both laboratories. Frequent updates (minimum: monthly) are recommended to ensure that records at both sites are complete. All data must be recorded.

4.5 Integrity of data used for reports, research publications and presentations

Any data used for reports, publications (including a thesis) and/or, presentation (e.g., abstract submission) must be clearly identified in one's laboratory notebook, with a summary of which experiments were used and any experiments that were excluded, with the reason (e.g., failed experiment – standard curve not acceptable). As part of organized record keeping, it is recommended to note where this information is summarized in each laboratory notebook, or in a separate laboratory notebook, if appropriate.

To ensure the integrity of data, the supervisor, or delegate, is required to verify that: 1) there are appropriate source data files for a manuscript, thesis, committee report and/or presentation, and that 2) the graphs, tables, and/or narrative description of findings, accurately convey the experimental findings, without bias. Both individuals (the record keeper and the individual verifying the records) must sign in the laboratory notebook whenever a data integrity review has taken place.

This SOP was developed in conjunction with the template provided by Parker and colleagues (Parker *et al.*, 2015).

REFERENCE

Parker D, Soomro A, Hayward CPM. Responsible Conduct and Documentation of Research: A Standard Operating Procedure Template that can be Customized. *2015*. http://hdl.handle.net/11375/19148 [Accessed 10 Oct 2021].

SOP 1a: Documentation for experimental endometriosis

Purpose
This SOP describes the minimal information that should be collected at necropsy of mice used for experimental endometriosis. Appropriate documentation of experimental research is critical for both interpreting, replicating, and publication of research data.

Information to be documented:

1. Date of Disease Induction
2. Person(s) performing the necropsy
3. Date of Necropsy
4. Age of mouse at necropsy.
5. Short description of the experimental protocol., including:
    - Method for disease induction (eg, injection site; type of tissue used)
    - ovariectomy (± oestrogen)
    - If animal was intact, record the cycle phase at euthanasia. All cycling mice should be euthanized in the same cycle phase to avoid cyclicity as a confounder.

Gross observations for each animal should be described in detail. Including:

1. Presence of lesions and lesion number
    - Lesion location, size, colour, and vascularity.
    - Lesion should be photographed in vivo with a ruler/callipers and/or a microscope that can measure the lesions. At times, lesions can be photographed once they are removed.

2. Describe how the lesions were preserved. (e.g., fixed, frozen).

3. Describe other tissues that are removed (e.g., blood, peritoneal lavage, uterus, ovaries, liver etc) and how preserved.
    - If weights of the mouse or any organ are obtained, this information should be included in the record.

4. Note any other pathology that is observed (e.g., the presence of adhesions, adhesion strength, adhesion location, or pyometra and if a photograph was taken).

SOP 1b: Participant identification and documentation

Purpose

Tissue collection for research purposes requires local IRB approval, patient informed consent, detailed documentation, and clear participant characteristic inclusion and exclusion criteria valid for the hypothesis to be tested. Inclusion and exclusion criteria will depend on the hypothesis to be tested and will inform eligibility. Depending on the characteristics and whether they are necessary to determine eligibility or to inform the experiment itself, details can be documented from medical record abstraction, a brief self-completed or interview formatted inclusion/exclusion criteria form or will require participant completion of a more detailed self-reported questionnaire.

Most commonly, chimeric models will exclude *a priori* tissue from participants with known infectious disease (e.g. HIV, Hepatitis C) or a history of cancer other than non-melanoma skin cancers. For endometriosis cases, the experimental design may demand inclusion of tissue from anyone with endometriosis or may require restriction defined by visualized lesion phenotypes or presenting symptoms. For samples collected by pipelle biopsy, women with an IUD are excluded. Women who are pregnant/trying to get pregnant are also excluded and a negative pregnancy test is required prior to collection of sample(s). For participants providing control tissue, considering the hypothesis is essential to determine inclusion and exclusion criteria. For example, for studies exploring immune markers or response, exclusion of those with autoimmune conditions may be required; for diagnostics discovery, those with gynaecologic conditions with symptoms also associated with endometriosis must be included; for aetiologic-omics, those with gynaecologic conditions with overlapping pathophysiologic pathways must be excluded, or they must be included with sample size sufficient to explore gynaecological condition heterogeneity.

Beyond eligibility of donors, participant characteristics must be noted to the extent possible. Variations between donors, even within the same group is to be expected. The previously published EPHect data collection tool may be helpful and can be found here: https://ephect.org/tools/patient-questionnaire

Participant characteristics to be documented

1. Age – ideally recorded as birth month and year
2. Menopausal status
3. For those still menstruating
    - Typical cycle length in days
    - Cycle regularity
    - Date of first day of last menstrual period
4. Exogenous hormone exposure
    - Time since last exposure – ideally record as month and year
    - Mode of exposure (oral, patch, IUD, injection, suppository, topical)
    - Formulation / brand
5. Endometriosis or adenomyosis status
    - Never diagnosed
    - Clinical diagnosis without visualized confirmation
    - Diagnosed via trans-vaginal ultrasound and/or MRI
    - Diagnosed via surgical visualization and/or hysterectomy
        i. Histologic confirmation
            1. Confirmed
            2. Disconfirmed / inconclusive
            3. No pathology report
6. Endometriosis Case Phenotypes
    - Time since diagnosis – ideally month and year
    - rASRM stage score and / or ENZIAN score and / or EFI score
    - Endometrioma yes / no
        i. Unilateral or bilateral? Size?
    - Deep endometriosis yes / no

- Presenting symptoms
    i. Time since symptom onset – ideally month and year
    ii. Infertility
    iii. Dysmenorrhea
    iv. Non-menstrual pelvic pain
    v. Dyspareunia
    vi. Dyschezia
    vii. Dysuria

7. Other gynecologic condition status
    - Ever diagnosed with uterine fibroids, pelvic inflammatory disease, endometrial polyps, PCOS, uterine fibroids, Asherman's Syndrome, simple ovarian cyst, endometrial hyperplasia, other
8. Other pain or immune condition status
    - Ever diagnosed with fibromyalgia, chronic migraines, irritable bowel syndrome or disorders, rheumatoid arthritis, SLE, Sjogren's syndrome, chronic fatigue syndrome, long COVID, Type I diabetes, multiple sclerosis, Parkinson's disease, grave's disease, and others
9. Endocrine disorder status
    - Ever diagnosed with an adrenal disorder, Type II diabetes, hypo- or hyperthyroid disorder, hyperprolactinemia, other

SOP 2: Universal precautions

Purpose
Human blood, tissues, and body fluids can be carriers of numerous infectious agents (e.g., HIV, Hepatitis B). All tissues/bodily fluids must be treated as though they are infectious, and universal precautions are therefore required. PPE (Personal Protective Equipment) is used anytime exposure to blood or other potentially infectious material is possible.

Minimum PPE required for the handling of human tissues include gloves, disposable gown, and shoes. Human tissues should be handled within a BSL2 biosafety cabinet. If tissues will be handled on an open bench, googles, and/or face shield is required.

PROTOCOL

1. Remove extraneous supplies (pipet tips, etc) from the hood. Wipe down hood with appropriate disinfection agent.
2. Place a beaker or other vessel containing 10% bleach in the hood to collect liquid waste (spent media, wash media, excess blood/mucous). *Contact time in bleach must be 10 minutes or longer.*
3. Change gloves if they become contaminated. Remove gloves regardless of perception of contamination whenever moving away from the hood or open bench. Remove gloves by peeling them away from wrist to fingertips.
4. When finished working with human samples, discard bleach/waste in an appropriate sink followed by copious amounts of water. Wash the beaker as appropriate.
5. Wipe down hood with appropriate disinfection agent.

SOP 3: Human endometrial tissue collection

Purpose

Xenograft models of experimental human endometriosis require acquisition of human tissues, preferably endometrial biopsies. Although some laboratories have successfully established experimental disease using ectopic lesions, current evidence indicates that the take rate is low and excised lesions frequently contain extraneous tissues. Therefore, this SOP refers **only** to eutopic endometrium collection. Endometrial biopsy requires a health care professional trained in the procedure with experience that confirms consistent safe collection and reliable yield of experiment-worthy tissue. Biopsies can be conducted during laparoscopic surgery or in an outpatient setting.

Inclusion criteria

1. Women with regular 25-35 day cycles, between 18 and 42 years
2. No hormonal medication (eg, oral contraceptives, contraceptive coil, progestogens, GnRH analogues) in 3 months prior to biopsy
3. Endometriosis status staged by rASRM and confirmed surgically OR healthy women without known reproductive disease. *Note: Numerous studies include patients with fibroids as a control population although this is not ideal.*
4. Stage of cycle based on last menstrual period should be recorded and confirmed histologically using Noyes Criteria. Collection of an endometrial biopsy is perhaps most commonly used; however, experimental disease has also been successfully established using secretory or menstrual tissues.

Exclusion Criteria

1. Endometrial pathology – polyps, hyperplasia, submucous fibroids, Asherman's syndrome
2. Endocrinological condition –adrenal disorder, diabetes, PCOS, thyroid disorder, hyperprolactinaemia
3. History or current malignancy
4. History of autoimmune disorders
5. Inability for informed consent

Note

These are general recommendations for inclusion/exclusion criteria which can be adjusted as appropriate.

Tissue Preparation

1. Tissues are typically provided to the laboratory in sterile PBS or Ringer's. Tissues can be kept at 4°C until use. Ideally, samples should be used within 12 hours. Tissues should be washed 2-3 times in sterile PBS to remove blood and mucous. If tissues have a lot of blood, they can be placed in Hank's Balanced Salt Solution in a shaking water bath to aid in its removal.

2. A portion should be placed in formalin for paraffin embedding and histological assessment. Stage of cycle based on last menstrual period should be recorded and confirmed histologically using Noyes Criteria (Noyes and Haman, 1953; Noyes *et al.*, 1975).

3. A portion can be frozen or placed in RNAlater® for molecular assessment.

4. Using sterile techniques, mince tissues into 1 mm^3 fragments (keep in PBS). Tissue fragments can be sorted into groups using a 24-well culture plate with each well containing tissues to be injected into a single mouse. **The amount of human tissue must be consistent between mice and between experiments using different human samples**. 8-10 one mm^3 fragments per animal is the recommended standard. Injection of fewer fragments will result in minimal disease. If tissues will be cultured overnight, place 10-12 pieces of tissue per mouse in culture since some loss will occur overnight. Tissues to be injected immediately should be kept in PBS. If they will be cultured, the appropriate media should be provided.

REFERENCES

Noyes RW, Haman JO. Accuracy of endometrial dating; correlation of endometrial dating with basal body temperature and menses. *Fertil Steril 1953*;4:504-517.

Noyes RW, Hertig AT, Rock J. Dating the endometrial biopsy. *Am J Obstet Gynecol 1975*;122:262-263.

SOP 4: Labelling of endometrial tissue

Purpose

If desired, human endometrial tissues may be labelled with a fluorescent dye prior to introduction into mice. Labelling aids identification of lesions at necropsy.

Supplies and equipment

- Sterile forceps.
- DMEM medium: [DMEM + 10% FBS with (1% P/S + Fungizone)] and without antibiotics.
- Blades of scalpel.
- 1 mL syringes and moderate bezel needles (18G-19G)
- Beaker with bleach.
- 96-well plates.
- Petri dishes.
- Fluorescent dye (e.g. AdCherry (Elution 1 (purified in UV) [1 · 1010 ifu / mL]).

PROTOCOL

Universal precautions (See SOP 2) should be used throughout this protocol.

1. Prepare all materials in a hood. Prepare a beaker with bleach to collect liquids that have come in contact with human tissues/samples. A second beaker with bleach should be used to collect pipet tips in contact with the adenovirus.
2. Following the acquisition of an endometrial biopsy, gently wash with sterile PBS. This can readily be accomplished by placing tissues in a 50 mL conical with saline and inverting multiple times. For samples with an excessive amount of mucous or blood, Hank's Balanced Salt Solution may be preferred over saline.
3. Transfer tissues to a sterile petri dish and select appropriate pieces with forceps and transfer to a container with complete DMEM medium (with antibiotics). Discard mucus and blood clots. If time is prolonged, maintain at 4°C.

Labelling protocol with Ad-mcherry (Detailed in (Martinez *et al.*, 2019)):

1. Using scalpel blades, mince the endometrial biopsy into pieces approximately 10-5 mm^3. Larger pieces will reduce penetrance of the adenovirus.
2. Once the biopsy is minced, fill a new petri dish with droplets of DMEM, no bigger than 50 µL, leaving enough space between the drops so that they do not stick together. Make as many as pieces of biopsy to be infected. **See Figure 1** below.

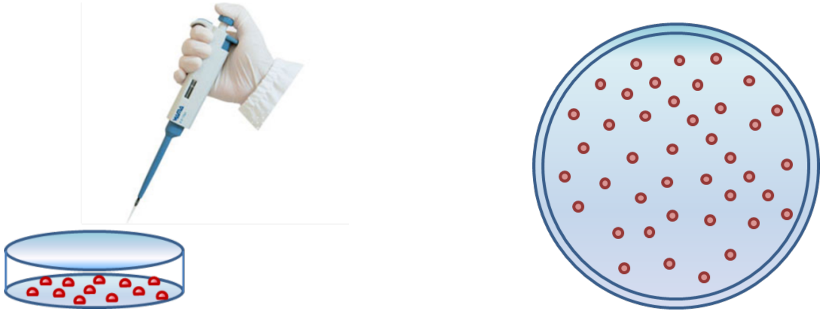

Figure 1. Droplets of DMEM.

3. Using a syringe with needle or wide bore pipet tip, aspirate one by one, endometrial sample pieces and transfer them inside each of the medium pearls at a 1: 1 ratio, so that each of the pearls contains a single piece of endometrium.

FROM THIS POINT, WEAR A DOUBLE PAIR OF GLOVES AND TAKE EXTRA CARE TO AVOID CONTAMINATING THE INNER OR OUTER SIDE OF THE HOOD.

4. Once the drops are filled, collect all unnecessary material and proceed to the preparation of the Ad-mCherry solution:
5. Prepare DMEM medium ***without*** antibiotics at a concentration 1:20 of purified elution 1 (stock should be kept at -80).
6. This will mean a volume of 10 µL of Ad-mCherry per 200 µL of medium.
7. (Pass pipette tips through bleach before discarding).
8. NOTE: The ideal dilution of the Ad-m-cherry stock for optimal labelling ranges between 1:10-1:40.
9. Dispense 200 µL of the Ad-mCherry solution per well of a 96-well plate (pass the tips through bleach before discarding).
10. Once the plate is ready, again with needle attached to a syringe, aspirate the tissue content of each pearl. *Minimize the transfer of media to avoid excess antibiotics*. Rinse with DMEM without antibiotics and transfer each tissue piece to a new well containing diluted Ad-m-cherry. (Remember to pass needles and syringe through bleach before discarding).
11. Check that all wells with medium contain endometrial sample and bring to incubator at 37°C and 5% CO_2.
12. *Culture tissues with ad-m-cherry medium between 16-24 hours.*
13. After the infection period, rinse tissues with fresh medium and transfer to a new 96-well plate filled with complete (with antibiotics) DMEM medium. (Remember to rinse all materials used in bleach before discarding)
14. Check for the labelling quality at the fluorescence microscope (λ: Red). Select and mark the wells that have been infected successfully, for their subsequent transplantation.
15. If assessment of tissue viability is required/desired, infected cells can be labelled also with CFSE (Carboxyfluorescein diacetate succinimidyl ester; only living cells are labelled), see images below.

IMAGES

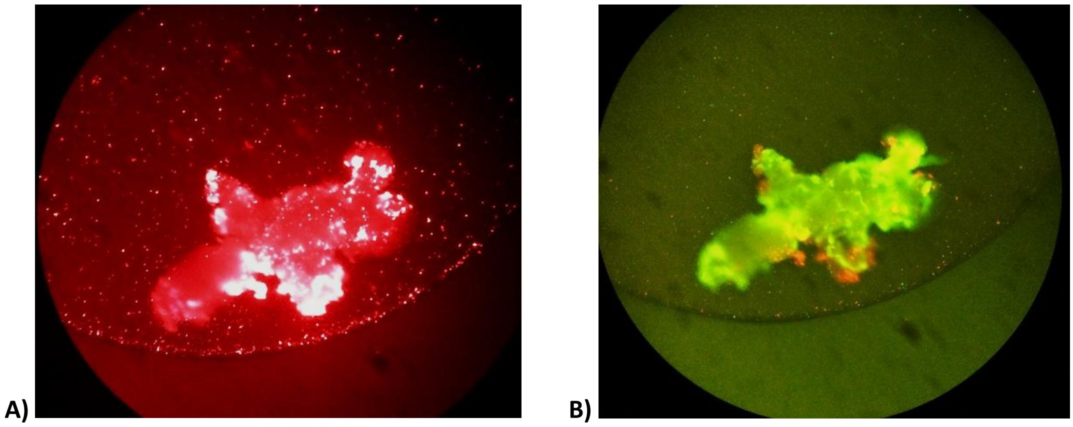

Figure 2: A) Microscopic image of endometrium infected with Ad-mCherry-E1 at 1:10 for 24 hours.
B) Microscopic image of the same infected endometrium, also marked with CFSE (viability test).

REFERENCE

Martinez J, Bisbal V, Marin N, Cano A, Gomez R. Noninvasive Monitoring of Lesion Size in a Heterologous Mouse Model of Endometriosis. *J Vis Exp 2019*.

SOP 5: Visual assessment of the murine cycle stage

Purpose

The mouse oestrous cycle is roughly 4-6 days in length. Oestrus typically lasts less than 1 day. Mice stop cycling during lactation except for one oestrus phase occurring 12-20 hours postpartum.

PROTOCOL

The stage of the murine oestrous cycle can be determined by the appearance of the vagina as previously described (Champlin *et al.*, 1973).

1. Gently grasp animal by the tail to view the vaginal area.
2. Note the appearance of the vaginal area and stage her according to the descriptions below.

**Dioestrus**	Vagina has a small opening. Tissues are dry and without wrinkles.
**Proestrus**	Vagina is gaping and the tissues are reddish-pink and moist. Numerous longitudinal folds or striations are visible on both the dorsal and ventral lips.
**Oestrus**	Vaginal signs are similar to proestrus, but the tissues are lighter pink and less moist, and the striations are more pronounced. Mice in oestrus are most receptive to mating.
**Metestrus-1**	Vaginal tissues are pale and dry. Dorsal lip is not swollen.
**Metestrus-2**	Vaginal signs are similar to metestrus-1, but the lip has receded. Whitish cellular debris may line the inner walls or partially fill the vagina.

Oestrous cycle staging can also be conducted by microscopic examination of cells collected by vaginal swabs (Zenclussen *et al.*, 2014). However, this is more time-consuming and daily assessments may induce stress, potentially disrupting cyclicity.

REFERENCES

Champlin AK, Dorr DL, Gates AH. Determining the stage of the estrous cycle in the mouse by the appearance of the vagina. *Biol Reprod 1973*;8:491-494.

Zenclussen ML, Casalis PA, Jensen F, Woidacki K, Zenclussen AC. Hormonal Fluctuations during the Estrous Cycle Modulate Heme Oxygenase-1 Expression in the Uterus. *Front Endocrinol (Lausanne) 2014*;5:32.

SOP 6: Ovariectomy and steroid capsule placement

Purpose

An enhanced take-rate of experimental disease has been noted in oestrogenised mice compared to cycling animals. This can be accomplished by performing ovariectomy and placement of a slow-release oestradiol (E2) capsule. Ideally, these procedures should be performed 2-5 days prior to tissue injection.

Supplies and equipment

- Sterile, slow-release in-house made E2 capsules (SOP 6a) or purchased E2 pellets (e.g. 17β-oestradiol Catalogue #E-121 [21 day release], SE-121 [60 day release] or NE-121 [90 day release]; Innovative Research of America, Sarasota, FL)
- Sterile iridectomy scissors
- Sterile rat tooth forceps
- Sterile small, blunt nose scissors
- Sterile mosquito haemostats
- Anaesthesia (e.g., isoflurane) and vaporizer (if appropriate)
- Analgesic (e.g., buprenex)
- Laminar flow hood
- Sterile field for surgery

Notes

Local institutional approval must be obtained prior to performing any procedure on a live mouse. Follow the recommendations of the analgesic of choice to achieve appropriate pain management as required (for example, the Association for Assessment and Accreditation of Laboratory Animal Care (AALAAC) recommends analgesia for abdominal surgery should begin prior to surgery and maintained for 48 hours after surgery).

PROTOCOL

1. Place the first animal in the vaporizer for anaesthesia. When she becomes unresponsive, move her to the surgical field in a prone position and place a nose cone so that she remains anesthetized. Shave the surgical area (if using haired mice) and swab with betadine followed by ethanol. Repeat betadine/ethanol swabs two more times.
2. Make a 5mm, dorsal incision through the skin of the flank of the mouse below the muscles surrounding the spinal cord. The incision should be centred between the bottom of the rib cage and the front of the hind-limb.
3. Gently separate the skin from the underlying muscle by inserting closed, blunt-nosed scissors between the skin and muscle and opening and closing the scissors to create a pocket. Confirm the correct location by locating the ovarian fat pad which should be visible under the muscle.
4. Using double sharp iridectomy scissors, the muscle fibres should be separated (not cut) by opening the scissors in a dorsal ventral direction. The incision should be held open with small rat tooth forceps and the ovary pulled out through the incision with blunt forceps by grasping the fat pad surrounding it. Mosquito haemostats should be placed at the boundary between the oviduct and uterus, and the oviduct ligated just below the haemostat. After removing the ovary and oviduct with iridectomy scissors, release the haemostat and inspect for bleeding. If bleeding is observed, replace the haemostat for several more seconds. Once bleeding has stopped, push the tip of the uterus back into the abdominal cavity.
5. Repeat the process on the other side using the same incision.
6. Insert a sterile oestradiol capsule or pellet under the skin using a trocar or creating a pocket:

   <u>With a trocar</u>: load trocar placing pellet in standing position on needle of trocar. Load obturator half-way into the trocar. Using your non-dominant hand, lift rodent's skin on the lateral side of the neck and insert trocar with your dominant hand. When the pellet in the trocar contacts the skin, twist trocar sideways, push through skin a distance of 2–3 cm towards the neck area. Push obturator to release pellet. Remove trocar.

   <u>Without a trocar</u>: Lift skin on lateral side of neck. Make an incision equal in diameter to that of the pellet to be implanted. Make a horizontal pocket using blunt tip forceps 2 – 3 cm beyond the incision site. Push the pellet into the pocket with the forceps towards the neck. Close the opening with wound clips.

Note: Skin sutures can be used but mice frequently bite them off.

7. Wound clips should be removed in in accordance with local animal care guidelines (typically, 8-14 days).

SOP 6a: Preparation of silastic capsules with oestradiol

Supplies and equipment

- Silastic tubing (ID 1/16; OD, 1/8) available from Fisher (#11-189-15G)
- Silicone type A medical adhesive (Dow Corning Health Care)
- Pharmaceutical grade oestradiol (Sigma E1024)
- Cholesterol (Sigma C8667)
- 2 small plastic petri dishes
- Vacuum with in-line filter
- Balance capable of measuring less than 1 gram.
- 1 mL pipet tips
- Cotton

This procedure should be performed in a Compounding Hood. If unavailable, a downdraft is acceptable. Wear gloves and a disposable lab coat. If using a downdraft, a mask is also recommended.

PROTOCOL

1. Wipe down area with 70% EtOH (ethanol).
2. Use a disposable pad to form a clean area within the hood.
3. Determine the number of capsules to be made. Cut the appropriate number of 1.2 cm lengths of silastic tubing. Place these in the top half of one petri dish and place in hood. Set the bottom half of the dish aside to hold completed capsules.
4. In the second petri dish, measure the appropriate amount of E2 (8 mg per capsule) and mix with cholesterol (75% E2; 25% cholesterol by weight). 150 mg E2 plus 50 mg cholesterol should produce ~16 pellets. Mix well.
5. Place the petri dish with E2 in a larger, secondary container within the hood. Place a pea-sized dollop of silicone in the lid and set aside. See **Figure 3** below for complete set-up.
6. Turn on vacuum and place a 1 mL pipet tip as shown in **Figure 4** below. Cover tip with small amount of cotton and place a silastic tube on tip of pipet tip. The cotton will prevent the majority of E2 from passing into the vacuum.
7. Once capsule is in place, vacuum steroid/cholesterol mix into the tubing. You may need to pack in the powder by tamping the tubing on the petri dish.
8. When filled, remove the tubing from the pipet tip and dip each end into the silicone. Wipe away excess with Kim wipes.
9. When all pellets are made, dispose of any leftover steroid by closing the petri dish and removing a glove over the dish. Place glove covered petri dish in biohazard trash. Change to new gloves and clean up. Wipe area with 70% EtOH.
10. Capsules should be stored at room temperature protected from light.

Sterilise capsules

Just prior to use in mice, capsules must be washed in PBS with antibiotics (eg, 1% antibiotic/ antimycotic mix) for 5 minutes followed by 2 washes of plain PBS. Capsules are now ready to use.

Capsules are expected to release steroid continuously for approximately 3 weeks. Based on the assessments by others (Suhonen *et al.*, 1993), it is predicted that 3.9 ng E2 will be released per day from each silastic capsule.

REFERENCE

Suhonen S, Sipinen S, Lahteenmaki P, Laine H, Rainio J, Arko H. Postmenopausal oestrogen replacement therapy with subcutaneous oestradiol implants. *Maturitas 1993*;16:123-131.

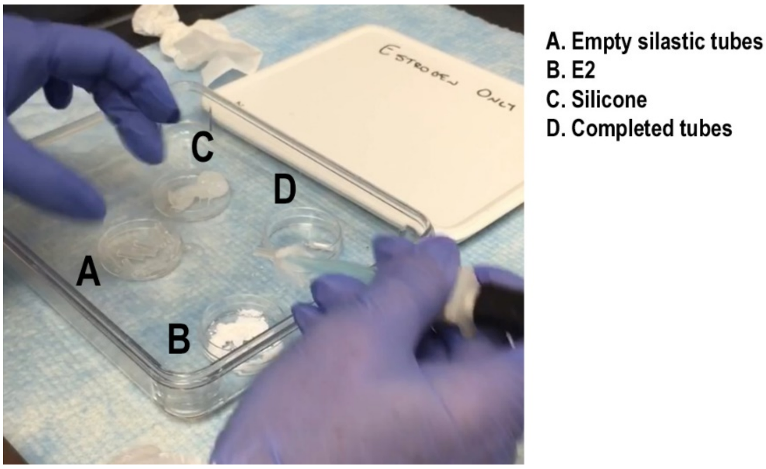

Figure 3: Recommended set-up for making steroid-filled silastic tubing.

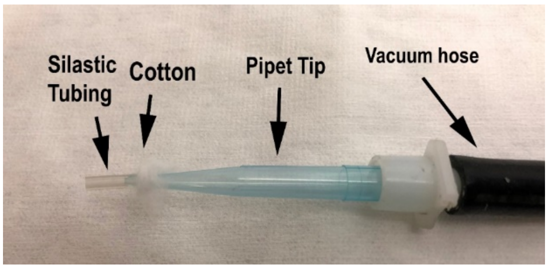

Figure 4: Recommended set-up for filling tubing with powdered E2.

SOP 7: Experimental human endometriosis in immunocompromised mice: Injection method

Purpose
To outline the standard operating procedures (SOP) for establishing experimental endometriosis in immunocompromised mice using human tissues.

Supplies and equipment
- Freshly acquired human endometrial tissues, minced and in sterile PBS (See SOP3)
- 5-6 wk-old immunocompromised mice (eg, athymic nude).
  - Mice may be used intact or ovariectomized and provided a slow release silastic capsule containing 8 mg oestradiol in cholesterol (see SOP6 and SOP6a). Premade slow-release pellets are also available from Innovative Research of America.
  - If using intact mice, it is preferable to establish disease during the oestrus phase. This is most simply achieved by providing a generous handful of bedding from a male's cage for 2 days prior to the procedure.
  - Mice should be provided sterile, low phytoestrogen rodent chow and water ad libitum.
  - Rooms should be maintained at ~26°C with a 12-hour light/12-hour dark cycle.
  - The weight of the animal should be checked prior to induction of disease, at least once per week after induction, and again at euthanasia.
- Sterile surgical instruments
  - blunt-tip forceps
  - sharp scissors if inserting a silastic capsule or pellet
  - 18-gauge needles (one per mouse)
  - tuberculin syringes (one per mouse)
- Sterile PBS
- 70% ethanol and betadine
- Sterile swabs
- Anaesthetic agent (e.g. Isoflurane) and vaporizer (as appropriate)
- Delta phase pad or heating pad (as recommended by local regulations)
- Sterile gloves and the paper insert saved for use as a sterile surgical field
  a. Alternatively, non-sterile gloves may be autoclaved
  b. Sterile paper should be used to cover the surgical field
- Laminar flow hood for surgery and preparation of tissues for injection

***Note**: If using ovariectomized mice, it is preferable to perform that surgery 2-5 days in advance (see SO 6).*

PROTOCOL

Important: The mice do not require an analgesic agent (e.g., buprenex) for tissue injection however, anaesthesia might be required for both comfort of the animal and safety to the human.

1. Human endometrial tissues must be washed twice in sterile PBS. After washing, using a sterile Pasteur pipette, load tuberculin syringes with tissues and at least 400 µL PBS. Attach needle and invert so that the needle is pointing down allowing tissues to settle near the needle.

   If tissues were cultured overnight, some loss of tissue integrity can be expected. At a minimum, each mouse should receive 8-10 mm^3 tissue fragments. PBS should be free of excess blood and mucous; however, *do not over wash tissues*. A slight yellow tint is ideal as cytokines produced by the tissues promote establishment of lesions.

2. After all syringes are loaded, place the first animal in the vaporizer for anaesthesia. When the animal becomes unresponsive, move her to the surgical field on her back and place a nose cone so that she remains anesthetized. If necessary, shave the area to be injected. Swab the abdominal area with betadine followed by alcohol (eg, 70%

isopropanol or 60% or higher EtOH). Repeat betadine/ethanol swabs two more times.

3. Loosen the cap of the appropriate syringe. Using the forceps, gently pinch the skin on the ventral midline to create a “tent”. Insert the needle, bevel up, into the tent. For subcutaneous injection, the needle should stay very close to the skin; for intraperitoneal (IP) injection, tilt the needle slightly toward the animal. Quickly eject the tissues and PBS. Remove the needle and pull back on the plunger to confirm all tissues were ejected. If tissues remain, draw up 200 µL PBS into the syringe and repeat the injection on the alternate side. Watch for bleeding (should be minimal).

4. After injection, immediately remove the mouse to a recovery cage. The cage should have a heating pad under *half* of the cage (this allows mice to move away from heat if desired). Observe that the mouse is awake and ambulatory before returning to the animal care facility.

   Lesions are well-established by 5 days post-injection.

SOP 8: Experimental human endometriosis in immunocompromised mice: Suture method

Purpose

To outline the standard operating procedures (SOP) for establishing experimental endometriosis by suturing human endometrial tissue into the peritoneal cavity of immunocompromised mice.

Supplies and equipment

- Freshly acquired human endometrial tissues, cut into fragments of 1.5 mm in diameter under sterile conditions, left in culture medium (DMEM Ham's F12 1:1) supplemented with Pen/Strep at 37°C and 5% CO_2 until transplantation. 4 tissue fragments per mouse.
- 5-6 wk-old athymic (nude) mice, rag2g(c) mice or (NOD)scid mice.
  - Mice may be used intact or ovariectomized and provided a slow release silastic capsule containing 8 mg oestradiol in cholesterol (SOP6 and SOP6a). Premade slow-release pellets are also available from Innovative Research of America.
  - If using intact mice, it is preferable to establish disease during the oestrus phase. This is most simply achieved by providing a generous handful of bedding from a male's cage for 2 days prior to the procedure.
  - c. Mice should be provided sterile, low phytoestrogen rodent chow and water ad libitum.
  - d. Rooms should be maintained at ~26°C with a 12 h light/12 h dark cycle.
  - e. The weight of the animal should be checked prior to induction of disease, at least once per week after induction, and again at euthanasia.
- Sterile surgical instruments
  - blunt-tip forceps
  - b. pointed-tip forceps
  - c. sharp small scissors
  - d. surgical suture material (one 5-0 suture and one 6-0 suture per mouse)
  - e. tuberculin syringes (one per mouse)
- Sterile clamps for mice (approximately 4-5 per mouse)
- Sterile PBS
- 70% ethanol and betadine
- Sterile swabs
- Anaesthetic agent (e.g. Isoflurane) and vaporizer (as appropriate), or Ketanest/Xylazin ip.
- Delta Phase Heating pad (or similar)
- Small sterile plastic petri dishes
- Ophthalmic ointment for mice
- Sterile gloves and the paper insert saved for use as a sterile surgical field
  - Alternatively, non-sterile gloves may be autoclaved
  - b. Sterile paper should be used to cover the surgical field
- Laminar flow hood for surgery and preparation of tissues for injection

PROTOCOL

1. Anesthetize the first animal (place in the vaporizer or IP injection of Ketanest/Xylazin). When she becomes unresponsive, move her to the surgical field in a back position. Apply some ophthalmic ointment to the eyes so that they do not dry out. Shave the surgical area (if using haired mice) and swab with betadine followed by ethanol. Repeat betadine/ethanol swabs two more times.
2. Immediately prior to transplantation, one human endometrial tissues fragment is clasped around with the end of a 6-0 suture thread, fasten the thread with a knot.
3. Make a 1 cm ventral incision through the skin in the midline of the belly of the mouse.
4. Gently separate the skin from the underlying muscle by inserting closed, blunt-nosed scissors between the skin and muscle, and opening and closing the scissors to detach the skin from the underlying skeletal muscles.
5. Using double sharp small or iridectomy scissors to open the muscles of the abdominal wall by a 5mm incision. Hold the cutting rim of the muscle layer by a forceps, while the needle of the surgical thread - at the end of which the tissue fragment is knotted - is inserted into the peritoneal cavity. Suture the fragment to the muscles of the

upper right quadrant of the abdominal wall from inside the peritoneal cavity at a distance of approximately 1 cm from the cutting edge. Take care that no internal organs are injured.

6. Perform the same procedure with the other 3 tissue fragments in the upper left, lower right, and lower left quadrant of the inner abdominal wall using the same incision.
7. Close the muscle incision with 4-6 surgical knots using a 5-0 surgical suture thread.
8. Close the skin incision with wound clips. Skin sutures can be used but mice frequently bite them off.
9. For pain relief after laparotomy, an analgesic (e.g., caprofen, 0.1 mg/20 g mouse) is administered subcutaneously.
10. After surgery, immediately remove the mouse to a recovery cage. The cage should have a heating pad under *half* of the cage (this allows mice to move away from heat if desired). Observe that the mouse is awake and ambulatory before returning to the animal care facility.
11. Wound clips should be removed in 8-10 days or consistent with local IACUC.

SOP 9: Retro-orbital injection of immune cells

Purpose
To outline the standard operating procedures (SOP) for retro-orbital injection of human immune cells. See SOP9a on Isolating Immune cells from whole blood.

Supplies and equipment
- 5-6 week-old rag2γ(c) mice or (NOD)scid mice previously or simultaneously established with experimental endometriosis (SOP 7 or 8).
- Sterile surgical instruments
  a. tuberculin syringes (one per mouse)
  b. 25 gauge needles (one per mouse)
- Anaesthetic agent (e.g. Isoflurane) and vaporizer (as appropriate)
- Ophthalmic anaesthetic
- Ophthalmic antibiotic ointment
- Sterile gloves and the paper insert saved for use as a sterile surgical field
  - Alternatively, non-sterile gloves may be autoclaved
  - Sterile paper should be used to cover the surgical field
- Laminar flow hood for surgery and preparation of cells for injection

Note: Ideally, immune cells should be injected 16-24 hours prior to tissue injection; however, depending on the purpose of the study, cells can be injected during the induction of experimental endometriosis.

PROTOCOL

Note: *<u>This procedure MUST BE PERFECTED using euthanized mice</u>. Only after demonstrating proficiency in dead animals should this procedure be attempted on live mice.*

1. Place the anesthetized mouse in left lateral recumbency with its head facing to the right and nosecone with aesthesia in place.
2. Partially protrude the mouse's right eyeball from the eye socket by applying gentle pressure to the skin dorsal and ventral to the eye.
3. Place a drop of ophthalmic aesthetic on the eye being injected.
4. Carefully insert the needle, bevel down, at an angle of approximately 30°, into the medial canthus. Placing the needle so the bevel faces down decreases the likelihood of damaging the eyeball. The operator uses the needle to follow the edge of the eyeball down until the needle tip is at the base of the eye. Slowly inject immune cells and then slowly withdraw the needle.
   a. -2.5 million immune cells per mouse in 100 µL PBS
5. Apply ophthalmic ointment to the affected eye.
6. Monitor the eye carefully to ensure that it has not been ruptured. Rupture of the eye requires immediate euthanasia.

SOP 9a: Isolation of PBMCs

Purpose

The purpose of this document is to outline the standard operating procedures (SOP) isolating peripheral blood monocytes plus neutrophils from patient blood samples.

Notes

- Ideally, cells should be isolated within 2 hours of blood collection, although PBMCs may remain viable for up to 8 hours.
- The most appropriate anticoagulant is EDTA, although others may be used.
- Work in strict conditions of sterility, both work area and materials.

Supplies and equipment

- BD Vacutainer™ tubes (with heparin or EDTA). 10 mL tubes, 3 or more tubes
- 19 gauge needle for blood collection
- Standard tissue culture supplies
- 1X PBS (room temperature)
- DF culture medium (DMEM/F12, phenol red-free)
- 0.2% (hypotonic) and 1.6% hypertonic NaCl solutions (ice cold) for RBC lysis
- 15 and 50 mL conical tubes
- Ficoll-Hypaque

PROTOCOL

Isolation of PBMCs:

1. Immediately after collection, tubes should be inverted 2-3 times.
2. Mix anti-coagulated blood 1:1 with sterile PBS. Set aside 2 mL for staining.
3. Place 15 mL Ficoll-Hypaque solution into a 50 mL tube and slowly layer the diluted blood over it by gently ejecting down the side of the tube. Maximum amount of blood mix is 35 ml. Alternatively, the Ficoll can be added under the blood by pipetting.
4. Centrifuge 20-30 min/400g at RT with brake off.
5. Using a Pasteur pipet, remove the upper plasma layer and discard. Carefully remove the buffy coat containing PBMCs (see **Figure 5**) and transfer to a new tube. Label “mononuclear cells”.
6. Remove and discard the Ficoll layer from the RBC pellet. Set aside the RBC pellet, which also contains PMNs.
7. Add ~40 ml DF to the mononuclear cell tube. Mix and centrifuge 10 min/400g/4°C.
8. Discard supernatant. If needed, lyse RBC by adding 20 mL 0.2% NaCl to pellet. Mix gently (slow vortex) 30 seconds. Immediately add 20 mL 1.6% NaCl. Mix and centrifuge 10 min/400g/4°C.
9. Discard supernatant and resuspend in 5 mL DF. Count cells and keep on ice until use.

Isolation of PMNs

1. Lyse RBCs from the PMN/RBC pellet by adding 20 mL 0.2 % NaCl. Mix gently (slow vortex) 30 seconds. Immediately add 20 mL 1.6% NaCl. Mix and centrifuge 6 min/250g/4°C. Repeat as needed (3x or more) until no RBCs remain.
2. Resuspend in 5 mL DF media. Count cells. Keep on ice until use.

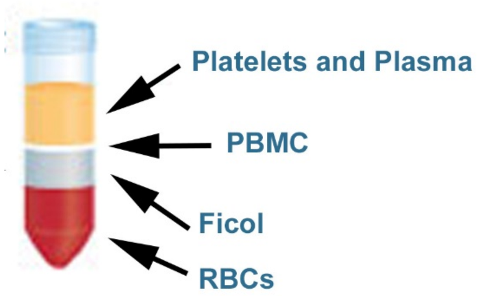

Figure 5: Identification of layers following separation of blood via Ficoll-Hypaque centrifugation.

SOP 9b: CFSE fluorescent labelling of PBMCs

Purpose
Labelling of immune cells prior to injection is useful to identify human cells residing in excised tissues.

Notes
- 10mM CFSE in DMSO (1000x).
- The CFSE can be handled with light, since it is not fluorescent by itself. Fluorescence is activated inside the cells and must be used in the dark after cells are labelled.
- The CFSE can be diluted with the media preferred by the cells to be marked; however PBS is recommended.
- Detection: Excitation = 492nm; Emission = 517nm.
- Recommended use concentration: 10 nM-10 µM, at this interval the CFSE is not toxic to the cells, even if increasing the incubation time.
- The quantity of fluorescence drops substantially during the first 24 hours, then it stabilizes and decreases depending on the cell division, so it is recommended to optimize the measurements of its activity at least 24 hours after the labelling.
- Work in strict conditions of sterility, both work area and materials.

Supplies and equipment
- CFSE Labelling kit
- PBS
- 15 mL tubes
- Pipette and tips of 1000 µL
- RPMI basic medium with and without antibiotics
- Vortex
- Aluminium foil
- Fluorescence microscope

PROTOCOL
1. Prepare CFSE at a final concentration of 10 µM in a final volume of 1 mL of PBS.
2. Resuspend the cells with 1 mL of RPMI basic medium without antibiotics. Gently mix by pipetting to separate individual cells. Transfer to a new 15 mL tube.
3. Carefully lay the open tube on its side. Do not contaminate the inside of the tube.
4. Add in the upper end of the tube (contrary to the suspension) diluted CFSE carefully avoiding any contact with the cells and close the lid**.**

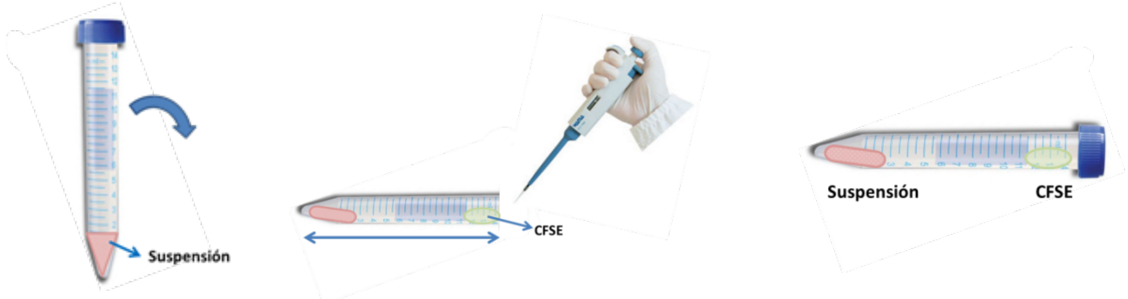

Figure 6: Tube position for Steps 3-5.

5. With the tube still in the horizontal position, replace the cap. Invert the tube and very quickly vortex (Figure 7) in order to achieve uniformity in the marking of the cells.

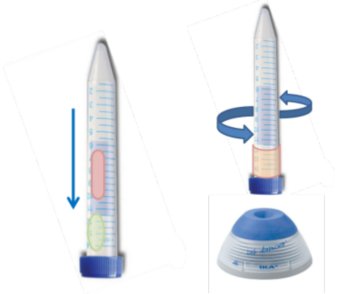

Figure 7: Cap the tube and vortex in the inverted position.

6. Cover the tube with aluminium foil to protect it from light and leave 15 min at room temperature.

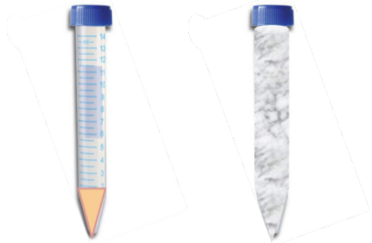

Figure 8: Cover the tube in foil to protect from light.

Note: *From this moment on, protect the cells from excess light to ensure fluorescence is not altered.*

7. Stop the labelling by adding the same volume of basic RPMI medium as reaction medium (Suspension + CFSE = 2 mL) and let stand 5 min (in darkness).
8. Centrifuge 5 min at 1600 rpm, room temperature.
   [YOU CAN OMIT THIS STEP IN CASE OF TISSUE RATHER THAN CELL LABELLING]
9. Remove and discard the solution of medium containing the CFSE and add another 2 mL of new medium to wash and remove the remaining CFSE.
10. Take an aliquot and check fluorescence under a microscope with a GREEN filter (Excitation = 492nm, Emission = 517nm).
11. Return the cells to their standard medium, in this case RPMI basic medium with antibiotics (to avoid contamination).
12. For culturing purposes, transfer cells to a flask and add the appropriate remaining medium. If cells are going to be injected into mice, transfer to a 1.5 mL Eppendorf tube.

SOP 10: Macroscopic assessment of lesions

Purpose
A standardized approach to the gross assessment of lesions at necropsy is critical for all studies and to compare studies across experiments and between laboratories. Although subcutaneous lesions are readily distinguished, histological assessment is often required for intraperitoneal lesions to confirm the endometrial origin of ectopic lesions. Additional assessments can be conducted using fixed or frozen samples, if desired.

Supplies and eqiupment
- Callipers or ruler
- Camera
- Several pair of forceps in various sizes
- Surgical scissors in various sizes
- Appropriate vessels for collection of lesions (e.g., cassettes and formalin for fixation, tubes for freezing, OCT media for preparation of tissue for cryosections)

PROTOCOL

Note: *If blood collection will occur at euthanasia, this should be done immediately after lavage.*

Although lesion collection will be more challenging after heart puncture or blood collection from the ascending aorta, rinsing the area thoroughly with room temperature PBS will facilitate this process.

1. Euthanise as approved by the local animal welfare committee.

2. Position the mouse dorsally on a disposable, absorbent cloth.

3. If desired, conduct a peritoneal lavage by injected 5 mL sterile PBS using an 18 g needle. With the needle still inserted, gently squeeze the abdomen to circulate the PBS. Slowly pull back the plunger to remove up to 3 mL of PBS. Set aside for analysis. Note—lavage cannot be conducted on mice that have undergone blood collection.

4. Next, carefully, remove the skin on the abdominal area without perforating the peritoneum. Inspect the peritoneum and opposing surface of the skin for lesions (see **Figure 9**). If found, measure with callipers or ruler. Photograph the lesion. Endometriotic-like lesion burden is scored by number and size, with size measured in two dimensions, the larger denoted “a” and the smaller denoted “b.” The total volume is calculated using the formula: V = a × b2/2. For lesions containing multiple distinguishable areas, separate measures may need to be taken and added together to determine the total lesion burden.

5. Carefully excise the lesion using the appropriate size scissors. Save as required for the intended analysis (e.g., formalin or frozen). Label the vessel appropriately.

6. Once the peritoneal surface has been inspected and lesions collected, remove the peritoneum and inspect the interior surface for lesions. Next, without disturbing any organ, visually inspect the opened abdominal cavity and note any abnormalities.

7. Using medium-sized forceps, gently grasp the large intestine and pull away from the body. Starting on the left side, carefully inspect each organ for lesions, adhesion, or other pathologies. Photograph and measure as needed. Carefully remove lesions and save in the desired medium.

8. For certain endpoints (e.g., testing of novel drugs), it may be appropriate to collect liver, spleen, and other tissues.

9. Once all lesions/tissues have been removed, the animal should be discarded as required by local regulations.

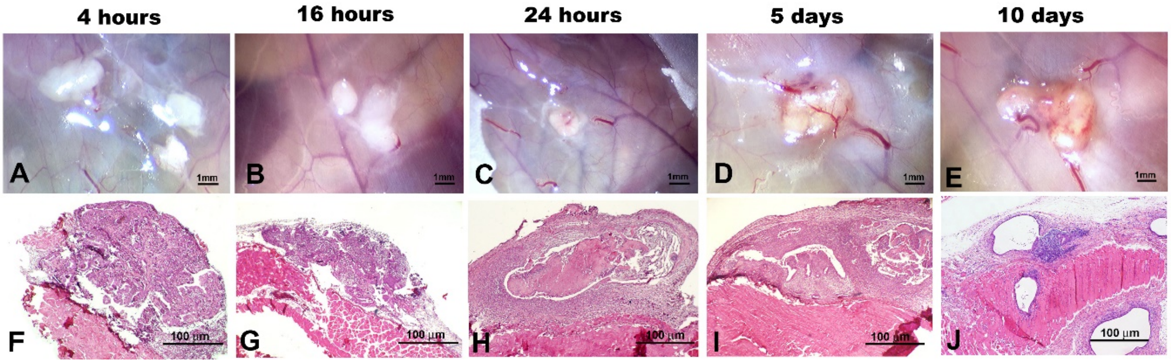

Figure 9: Gross (A–E) and microscopic (F–J, haematoxylin and eosin staining) photomicrographs of experimental endometriosis established by proliferative phase human endometrium in nude mice*. All mice were implanted with a slow-release oestradiol capsule before introduction of human tissues. Mice were killed 4 h to 10 d after human tissue injection. Results are representative of three separate experiments using three different human biopsies. Original magnification: gross, ×15; microscopic, ×40. (Bruner-Tran et al., 2009).*

REFERENCE

Bruner-Tran KL, Osteen KG, Duleba AJ. Simvastatin protects against the development of endometriosis in a nude mouse model. *J Clin Endocrinol Metab 2009*;94:2489-2494.
