# Supplementary material for: WERF Endometriosis Phenome and Biobanking Harmonisation Project for Experimental Models in Endometriosis Research (EPHect-EM-Heterologous): heterologous rodent models
Source: Mol Hum Reprod. 2025 Jul 9;31(3):gaaf022. doi: 10.1093/molehr/gaaf022 (PMC12237513; doi:10.1093/molehr/gaaf022)
Supplement: gaaf022_Supplementary_Data [file gaaf022_supplementary_data.zip › MHR-24-0371-R2-Supplementary info.pdf]

**Supplementary Information**

# World Endometriosis Research Foundation EPHect Experimental Models for Endometriosis Research (EPHect-EM-Heterologous): heterologous rodent models

M. Louise Hull, Raul Gomez, Warren B. Nothnick, Ruth Gruemmer, Katherine A. Burns, Mohammed Zahied Johan, Isabella R. Land, Stacey A. Missmer, Lone Hummelshoj, Erin Greaves, Kaylon L. Bruner-Tran, for the EPHect Experimental Models Working Group

Supplementary Figure S1: Timeline and framework of EPHect initiative.

Supplementary File S1: SOPs for EPHect Standard Operating Procedure Experimental models: heterologous (provided as a separate file)

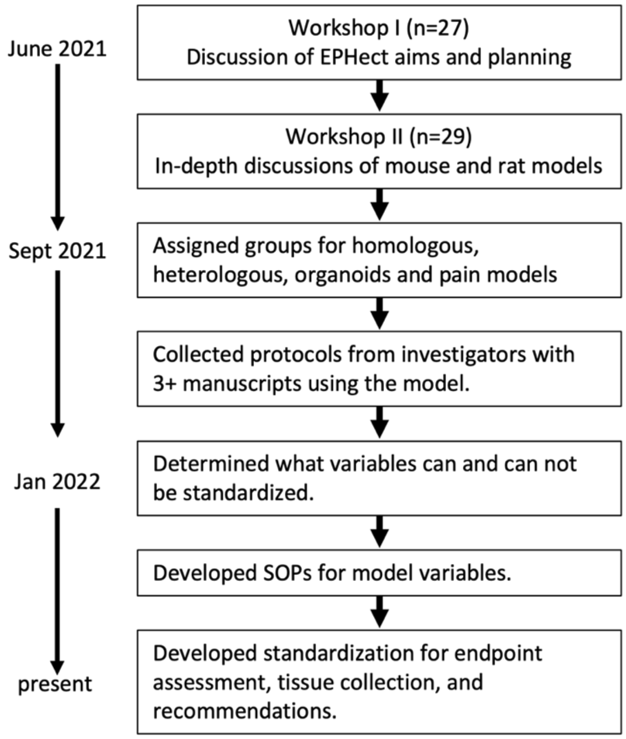


**Supplementary Figure S1: Timeline and framework of EPHect initiative.**
